# Supplementary material for: A joint proteomic and genomic investigation provides insights into the mechanism of calcification in coccolithophores
Source: Nat Commun. 2023 Jun 23;14:3749. doi: 10.1038/s41467-023-39336-1 (PMC10290126; doi:10.1038/s41467-023-39336-1)
Supplement: Supplementary file 1 — Supplementary Information [file 41467_2023_39336_MOESM1_ESM.docx]

**A joint proteomic and genomic investigation provides insights into the mechanism of calcification in coccolithophores**

Alastair Skeffington^1,2^, Axel Fischer^1^, Sanja Sviben^1^, Magdalena Brzezinka^1^, Michał Górka^1^, Luca Bertinetti^3^, Christian Woehle^4^, Bruno Huettel^4^, Alexander Graf^1^, André Scheffel^1,5,*^

^1^Max-Planck Institute of Molecular Plant Physiology, Potsdam-Golm 14476, Germany

^2^Biological and Environmental Sciences, University of Stirling, Stirling, FK9 4LA, UK

^3^Max-Planck Institute of Colloids and Interfaces, Potsdam-Golm 14476, Germany

^4^Max Planck Institute for Plant Breeding Research, Max Planck-Genome-centre Cologne, Cologne 50829, Germany

^5^Technische Universität Dresden, Faculty of Biology, 01307 Dresden, Germany

*Corresponding author: andre.scheffel@tu-dresden.de

# **Supplementary Figures**


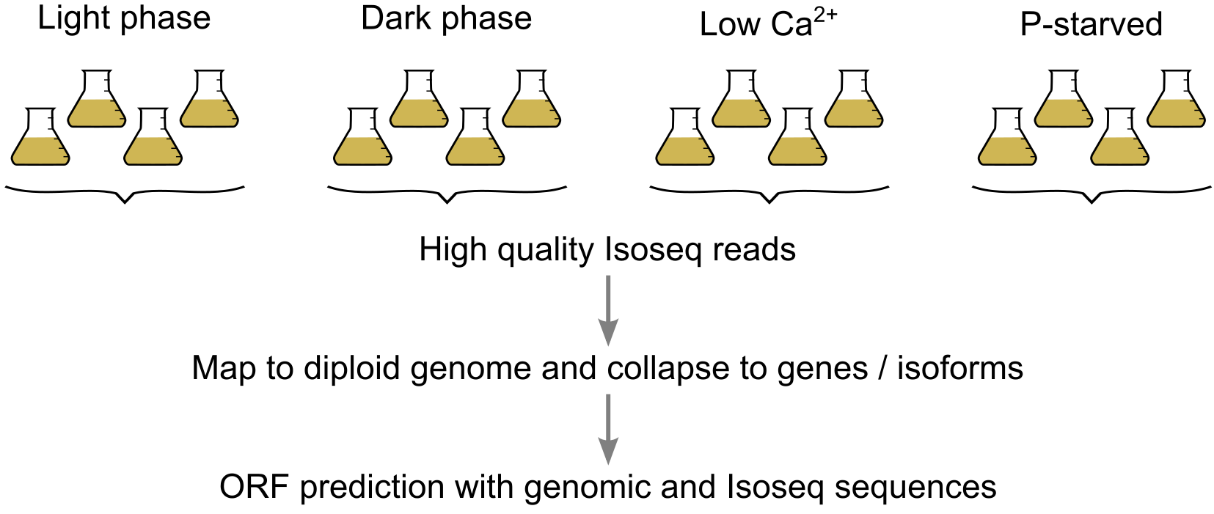


**Supplementary Figure 1: Experimental design for generation of *E. huxleyi* long-read transcriptome.** To obtain good coverage of the transcriptome and the repertoire of transcript isoforms, RNA was isolated for Isoseq sequencing from cells that had been (A) 6 h into the light phase and (B) 6 h into the dark phase in Ca-replete medium, (C) 6 h into the light phase in low-Ca medium, and (D) 6 h into the light phase after two days in zero-P-medium. Four independent biological replicates were pooled for sequencing post RNA extraction and quality control. For details on RNA extraction, library preparation, PacBio IsoSeq sequencing, transcriptome assembly and ORF prediction see Supplementary Note 1,2 and materials and methods.


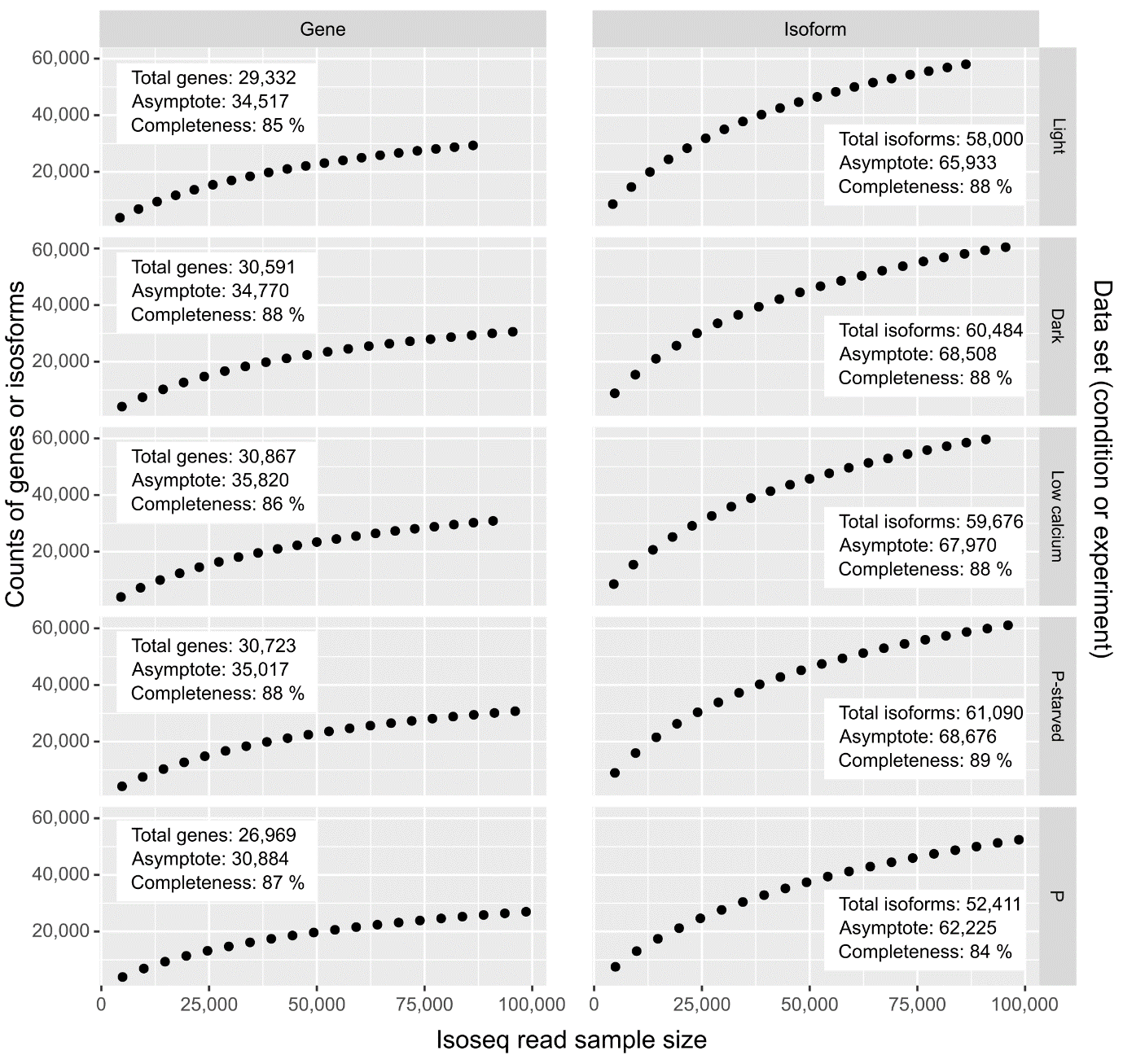
**Supplementary Figure 2: Estimation of completeness of Emihu2 at the gene and isoform level.** Rarefaction curves were generated by randomly sampling PacBio Full Length Non-chimeric reads and counting the number of genes and isoforms resulting from the selected reads. Asymptotes were calculated using SSasymp{stats} in R. Conditions: P = Preliminary data sequenced using the PacBio RSII system, derived from calcifying cells harvested in the light phase.

**
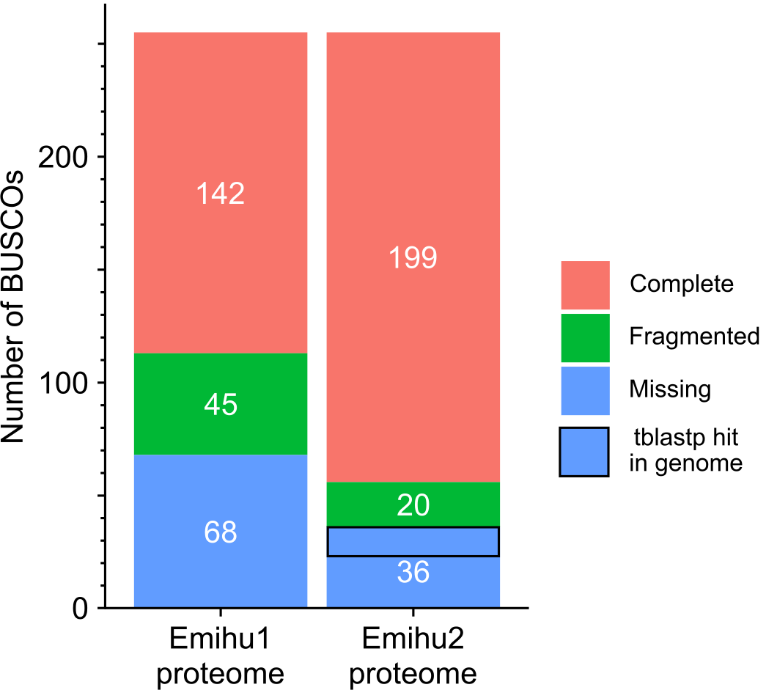
**

**Supplementary Figure 3: Completeness of the Emihu1 and Emihu2 proteome.** Eukaryote level protein BUSCO analysis (v4.0.5, based on the eukaryote_odb10 orthogroup set) of the Emihu2 proteome. Of missing BUSCOs, 12 have a possible homologue not found by the BUSCO analysis (BlastP e-values ranging from 0.0 to 3E-12, see Supplementary Table 2).

**
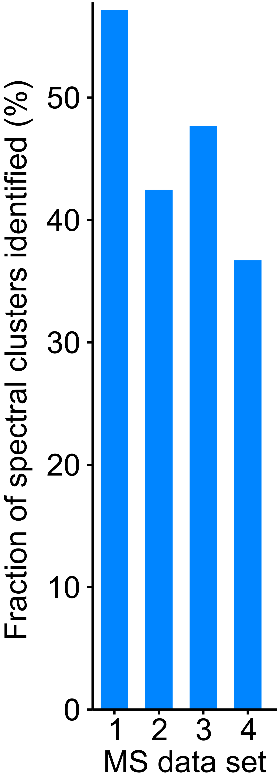
**

**Supplementary Figure 4: Identification rates of spectral cluster for an *Arabidopsis* proteomics dataset.** For information on the datasets see Supplementary Note 3.

**
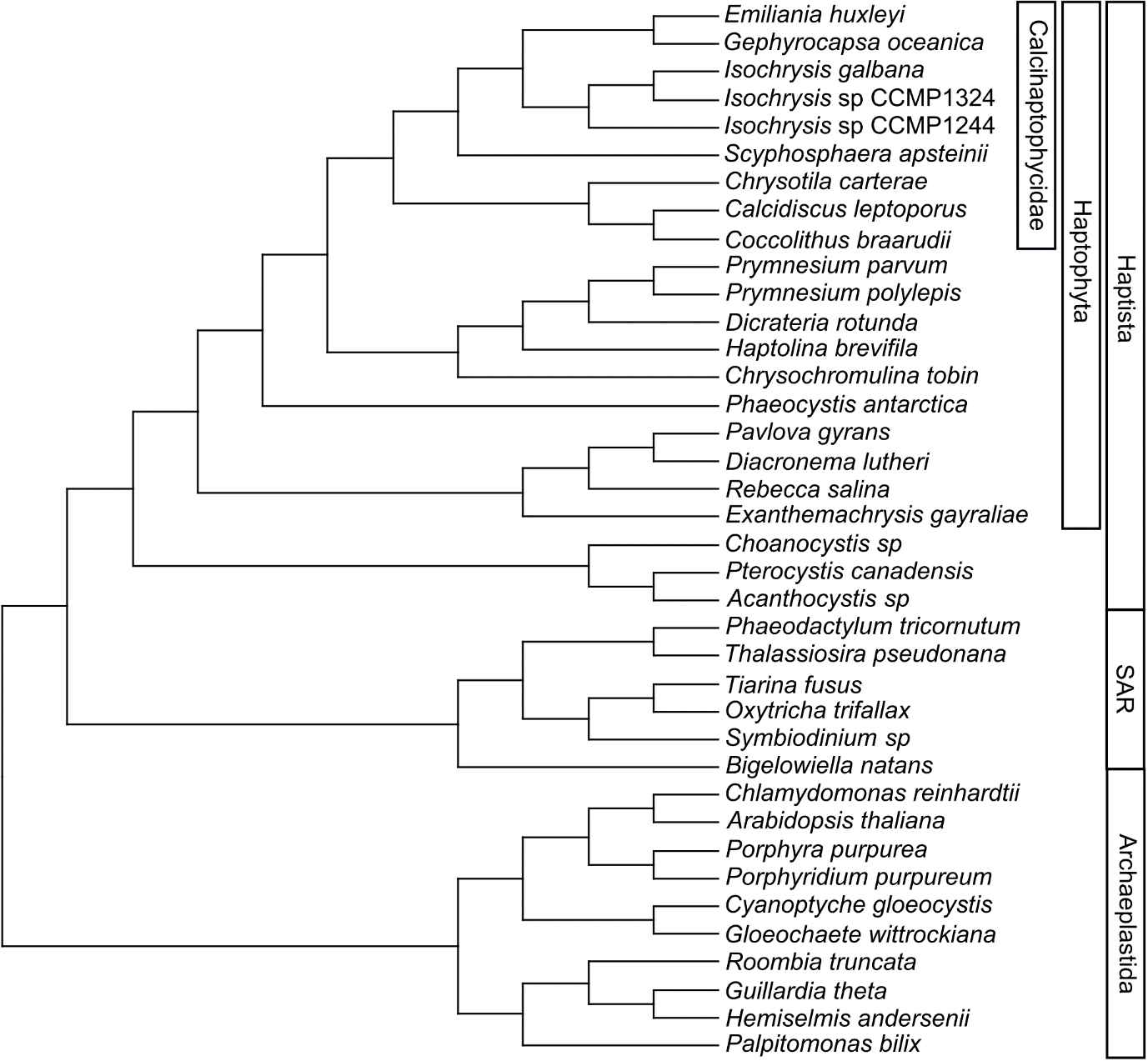
Supplementary Figure 5: Guide tree use for phylostratigraphic analysis.** For further species details refer to Supplementary Table 3. The tree is based on the latest Eukaryotic phylogenies^1^. Information to correctly place haptophyte species on the tree was taken from several sources^2–4^.


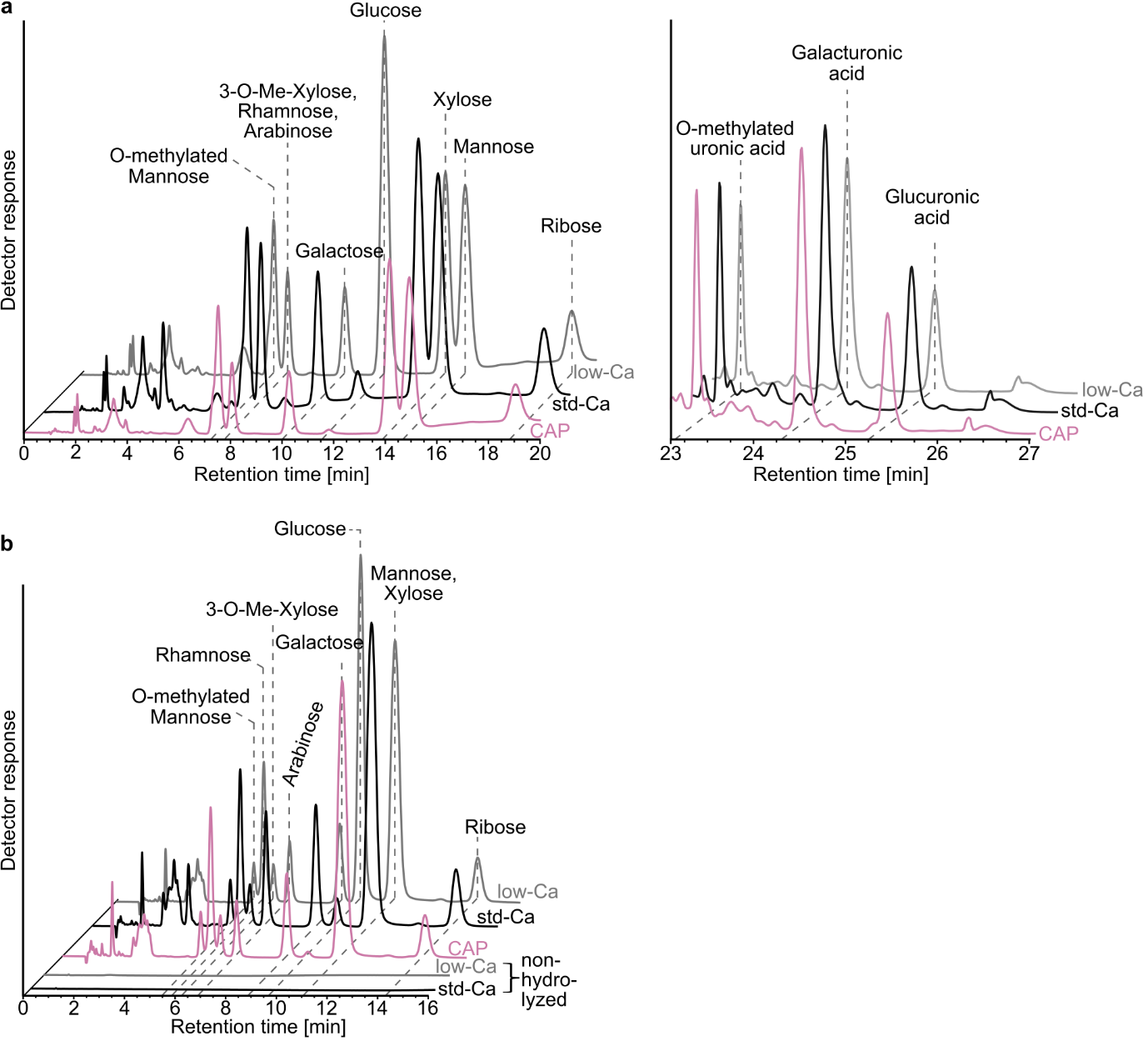
**Supplementary Figure 6: HPAEC-PAD analysis of the monosaccharides in hydrolyzed, EDTA-solubilized extracellular and coccolith associated material of *E. huxleyi*.** Chromatographic separation was carried out at 30 °C using a CarboPac20 guard column and IonPac CarboPac20 analytical column connected in series. (**a**) Chromatogram of the neutral sugars (left) and uronic acids (right) using a multi-step elution protocol, starting with 2 mM NaOH at the start of the analysis. This elution protocol allows the separation of mannose and xylose and their quantification. (**b**) Chromatogram of the neutral sugars using a multi-step elution protocol, starting with 10 mM NaOH at the start of the analysis. This elution protocol allows the separation of rhamnose and arabinose and their quantification. The absence of peaks in the chromatograms of the non-hydrolyzed samples shows that the monosaccharides in the hydrolyzed samples are not represent secreted monosaccharides but are of polysaccharide origin. Note that glucose in the CAPs hydrolysate was below the concentration range for quantification, and that the methylated monosaccharides could not be quantified due to technical difficulties.

**
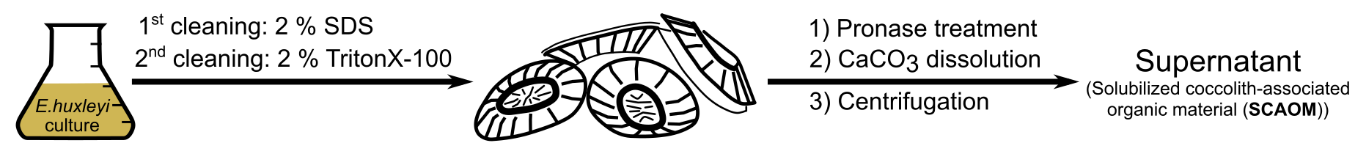
Supplementary Figure 7: Experimental design for the isolation of coccoliths and soluble coccolith-associated organic material (SCAOM) for biochemical and proteomic analysis.**


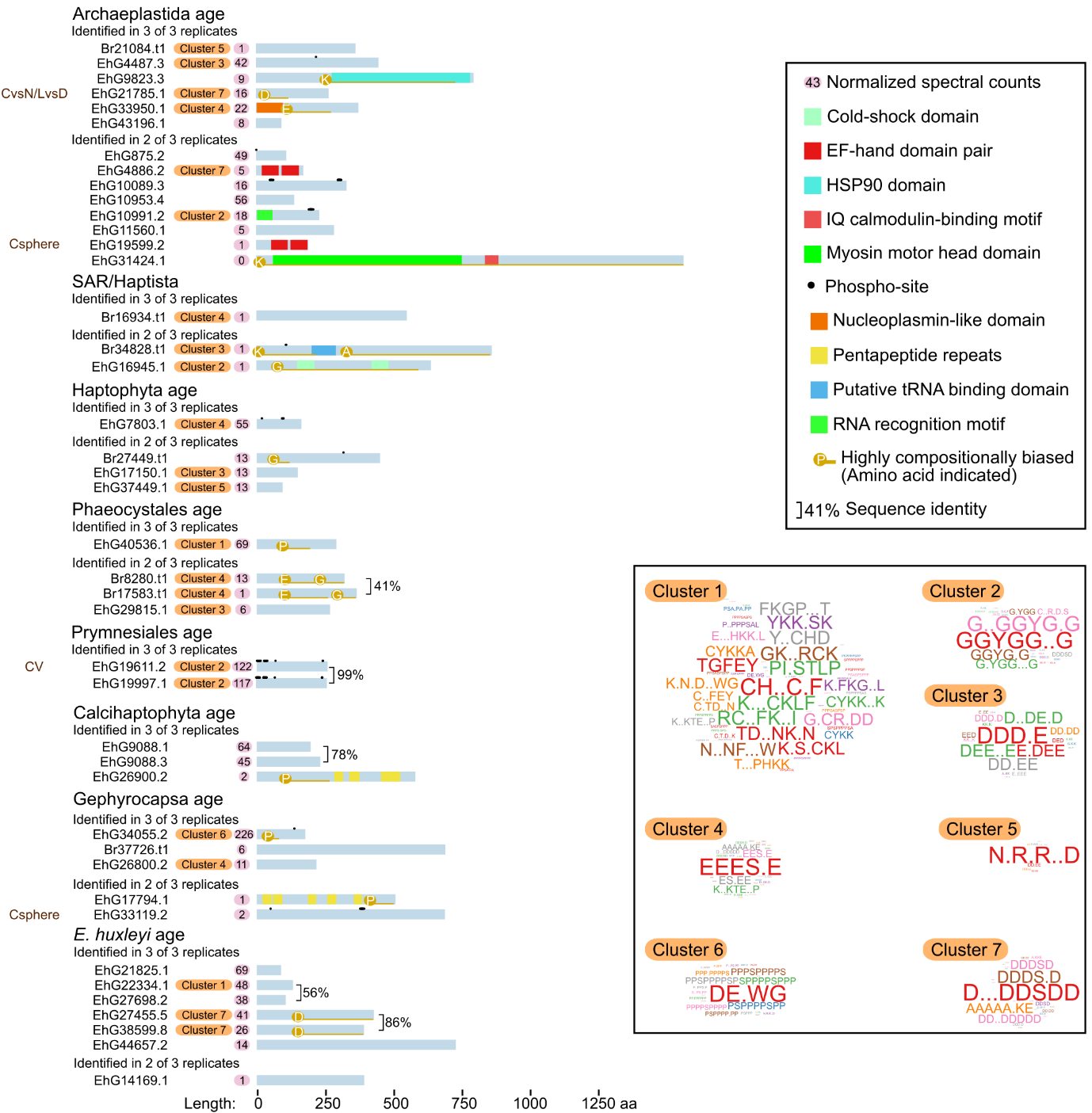
**Supplementary Figure 8: Graphical summary of all identified in the proteomic analysis of coccolith without known targeting motifs or with incomplete N-termini.** Proteins were assigned to clusters based on their shared motif contents using ProminTools^5^. The motif content of each cluster is described as a word cloud, where the size of the motif is proportional to its enrichment in that cluster compared to the predicted proteome as a whole. For the most similar sequences within the dataset, pairwise similarity in global sequence alignments is indicated by brackets. Proteins are organized by gene-age based on the phylostratigraphic analysis. To the left, the other datasets in which this protein is found are indicated: CSphere = Coccosphere, CvsN = C-cells vs N-cells, CV = coccolith vesicle, LvsD = Light vs dark.


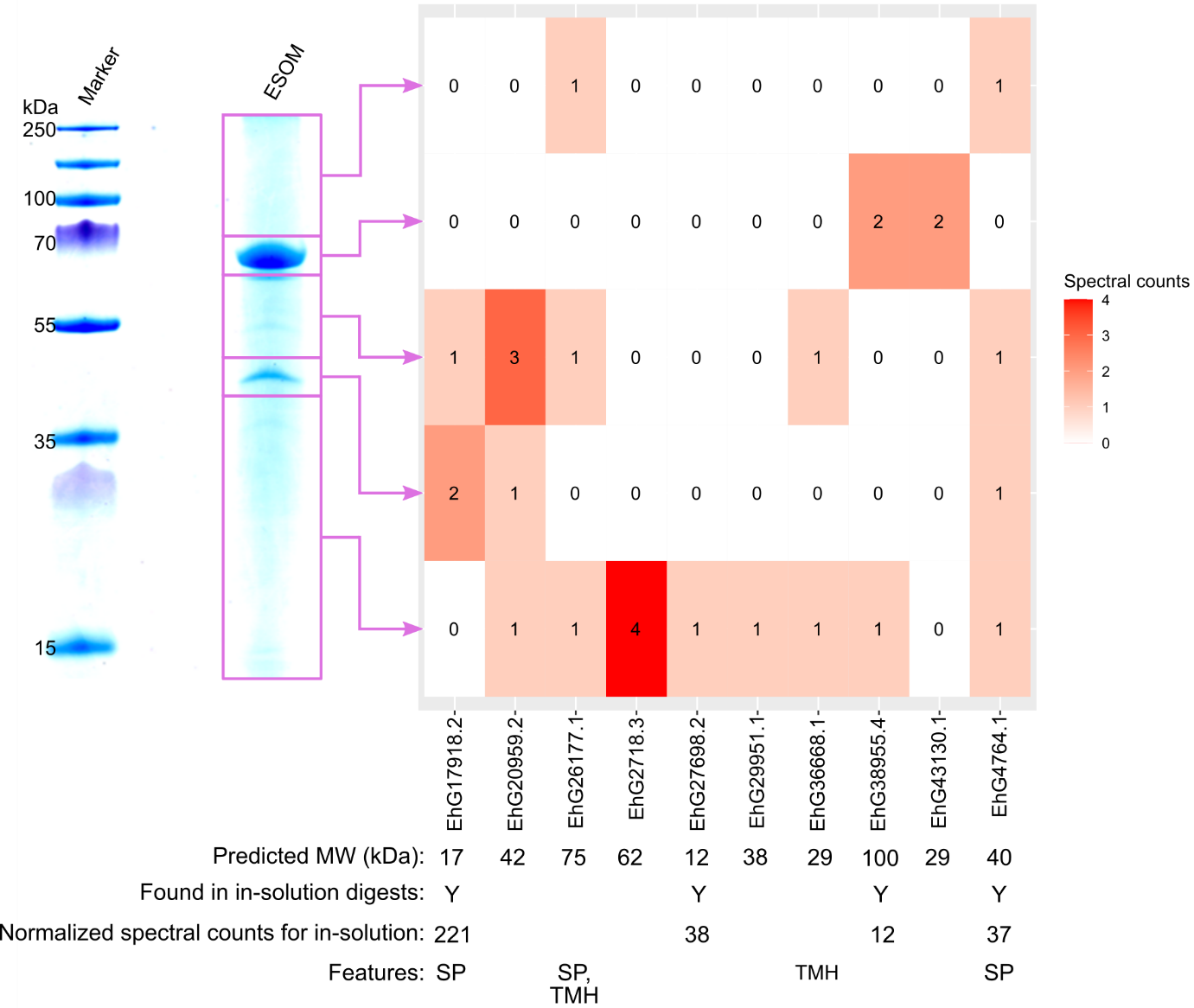
**Supplementary Figure 9: Proteomic analysis of bands cut from gel of deglycosylated ESOM.** An SDS-PAGE gel stained with Coomassie stain is shown left, and the lane on which ESOM was separated was cut into five pieces (fractions) as indicated. In each fraction, the number of spectra identified for each protein is shown as a heatmap. If also identified in the in-solution digests, this is indicated underneath the heatmap. Molecular weights are given without signal peptide if present. For schematic primary structures see figure S8. The results of SDS-PAGE gel analysis were the same in 3 independent biological replicates. For protein identification, the proteomic data of the same gel fraction from all samples were combined to generate the protein list for this gel fraction. This was necessary because the proteins in the gel pieces were poorly digested and only sparse MS data were available. SP: N-terminal signal peptide; TMH: Transmembrane helix.

**
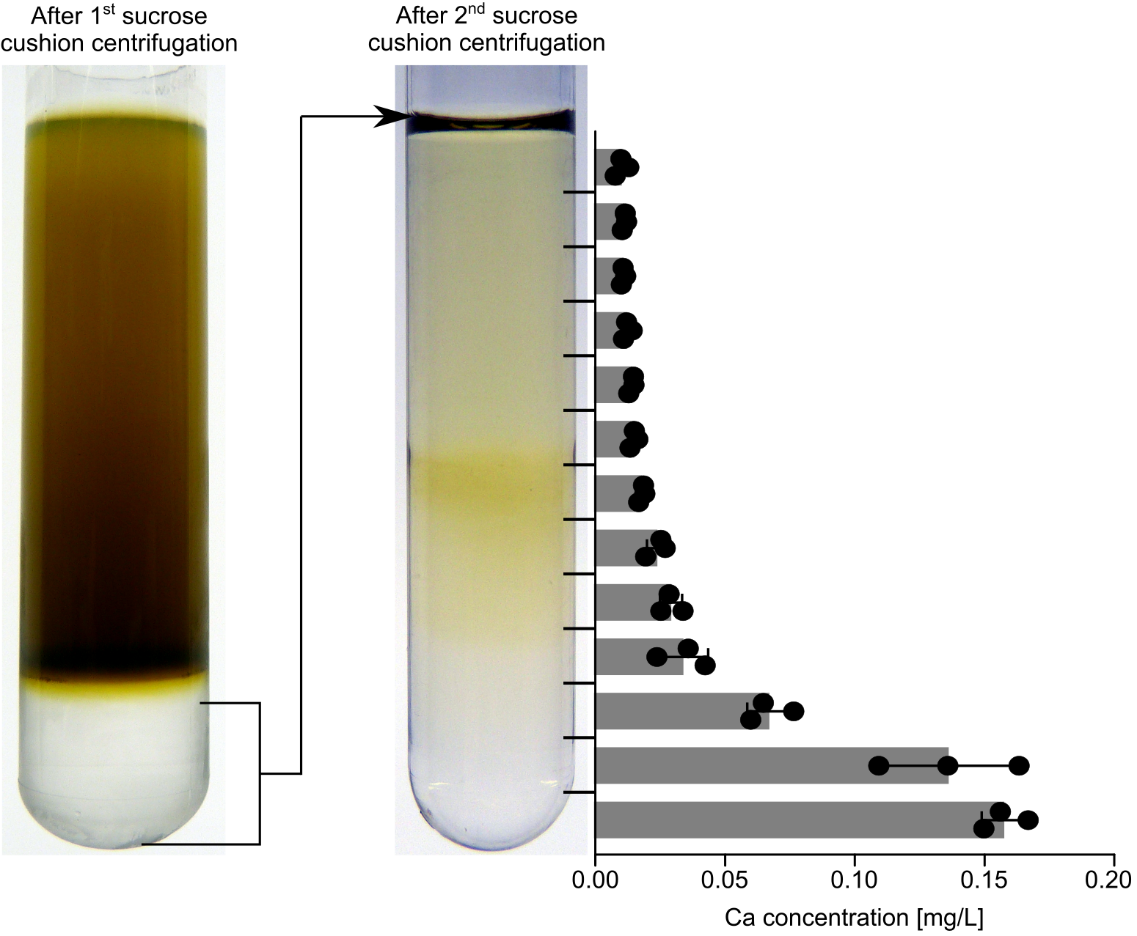
Supplementary Figure 10: Isolation of proto-coccoliths.** Cells whose coccolith shell had been removed were lysed using a French press, and unbroken cells and aggregated material were pelleted by low-speed centrifugation. The released intracellular nascent coccoliths were isolated from the cell homogenate by two successive rounds of centrifugation through a sucrose cushion. After the first round of centrifugation, the cushion was recovered, diluted with buffer and loaded onto the second sucrose cushion. After the second round of centrifugation, the tube content was fractionated. Quantification of the calcium by inductively coupled plasma optical emission spectroscopy (ICP-OES) revealed that the coccoliths had accumulated in the lower fractions. Data are represented as mean ± SD (n = 3, n = independent biological replicates). Source data are provided as a Source Data file.


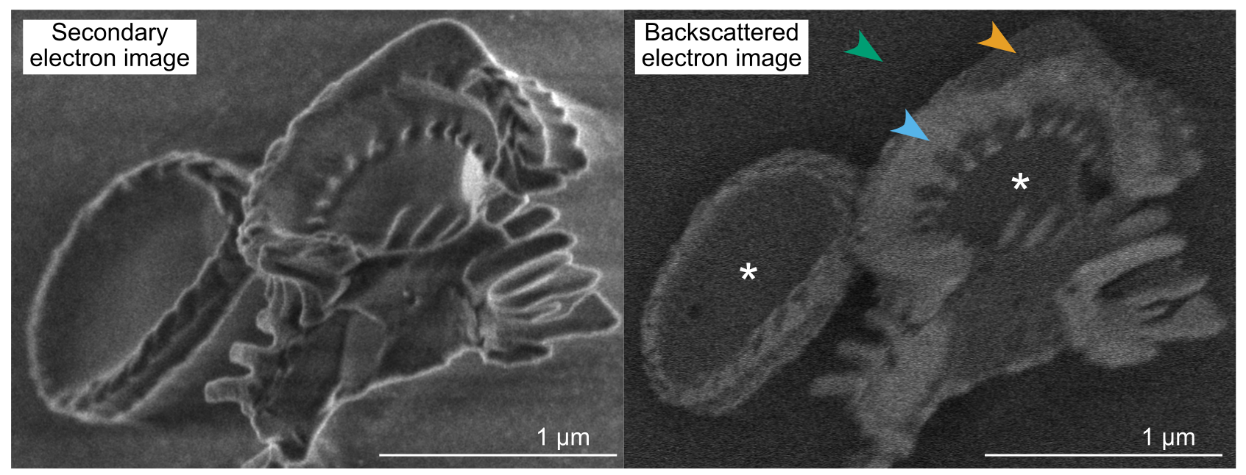
**Supplementary Figure 11: A coat of organic material encloses the calcite of isolated proto-coccoliths.** CryoSEM micrographs of freeze-fractured proto-coccoliths (n = 2, n = independent biological replicates). Images were acquired with an acceleration voltage of 1.2 kV and the semi-in-column detector, with the r-filter set to BS mode. Monte Carlo simulations on organic material with a density comparable to lipid bilayers and for 10^6^ electrons revealed that 95 % of the BSE with an energy of >1 kV, which were imaged, originated from a depth of about 5 nm. The backscatter image shows material (orange arrowhead) that has a brighter contrast than ice (green arrowhead) but lower contrast than calcite (bluish arrowhead), enclosing the mineral phase of proto-coccoliths. This material is also filling the central area (asterisk) of the coccoliths, which is not yet closed by calcite in early stage proto-coccoliths. The material enclosing the calcite is therefore very likely organic in nature and may represent coccolith vesicle membrane.


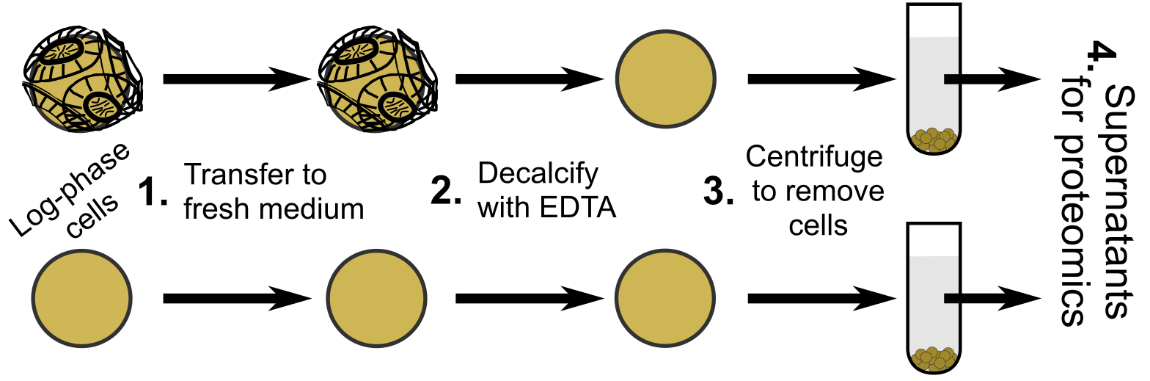
**Supplementary Figure 12: Experimental setup for the identification of coccosphere associated proteins.**

**
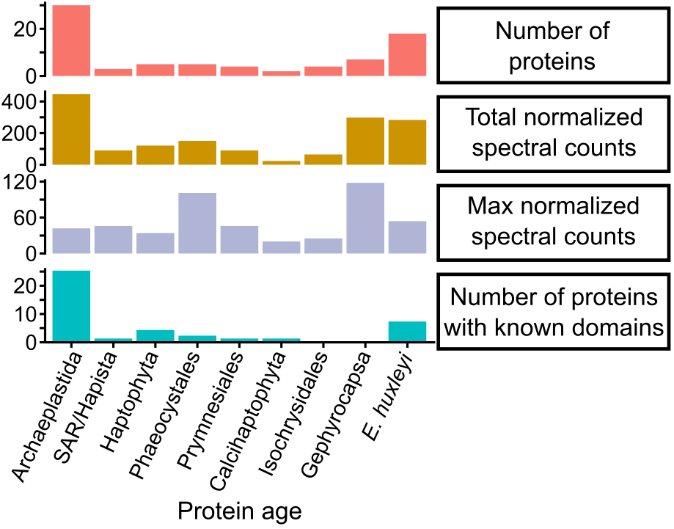
Supplementary Figure 13: Properties of the coccosphere dataset for proteins in each age category.**


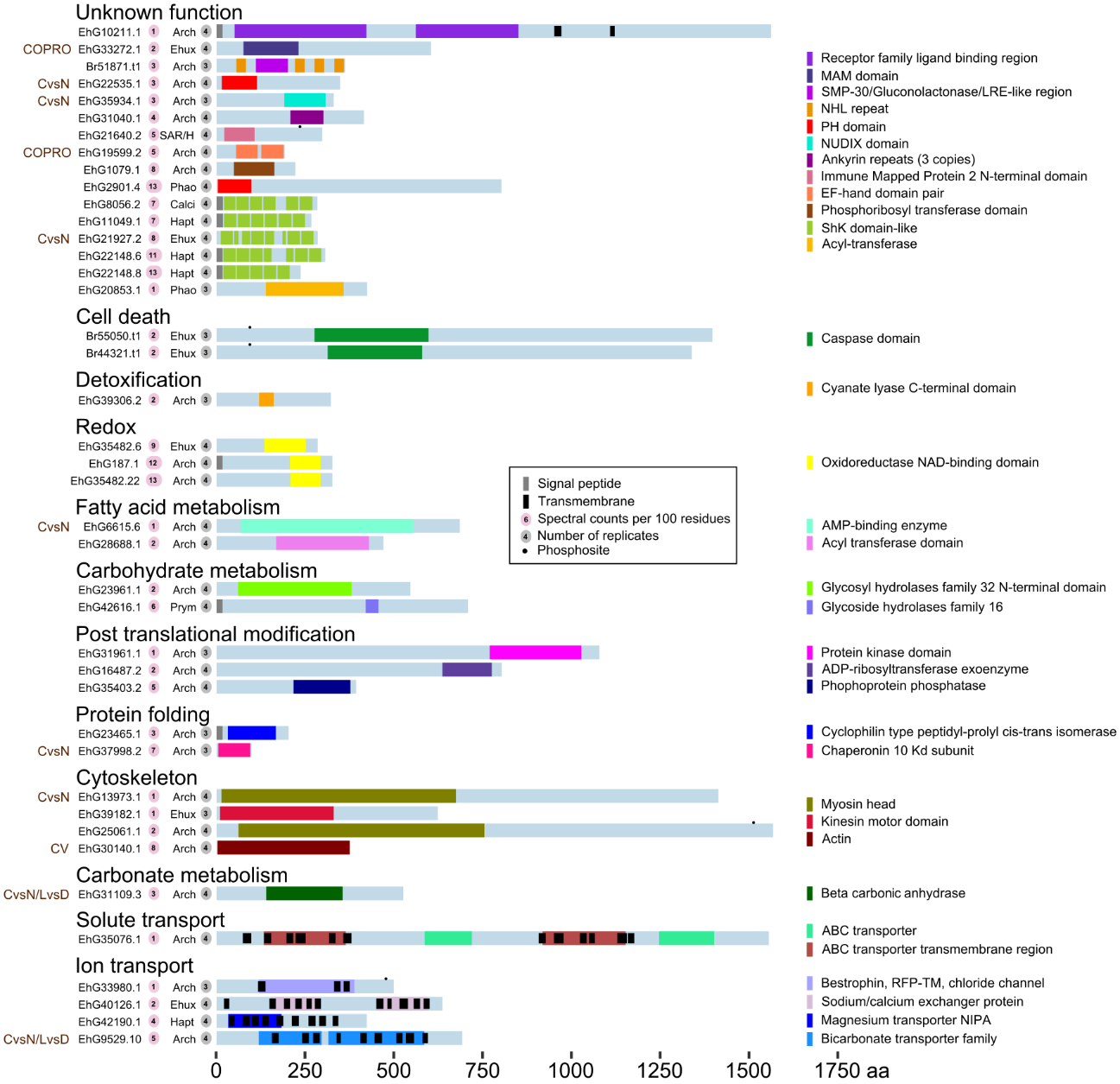
**Supplementary Figure 14: All coccosphere proteins with known domain.**


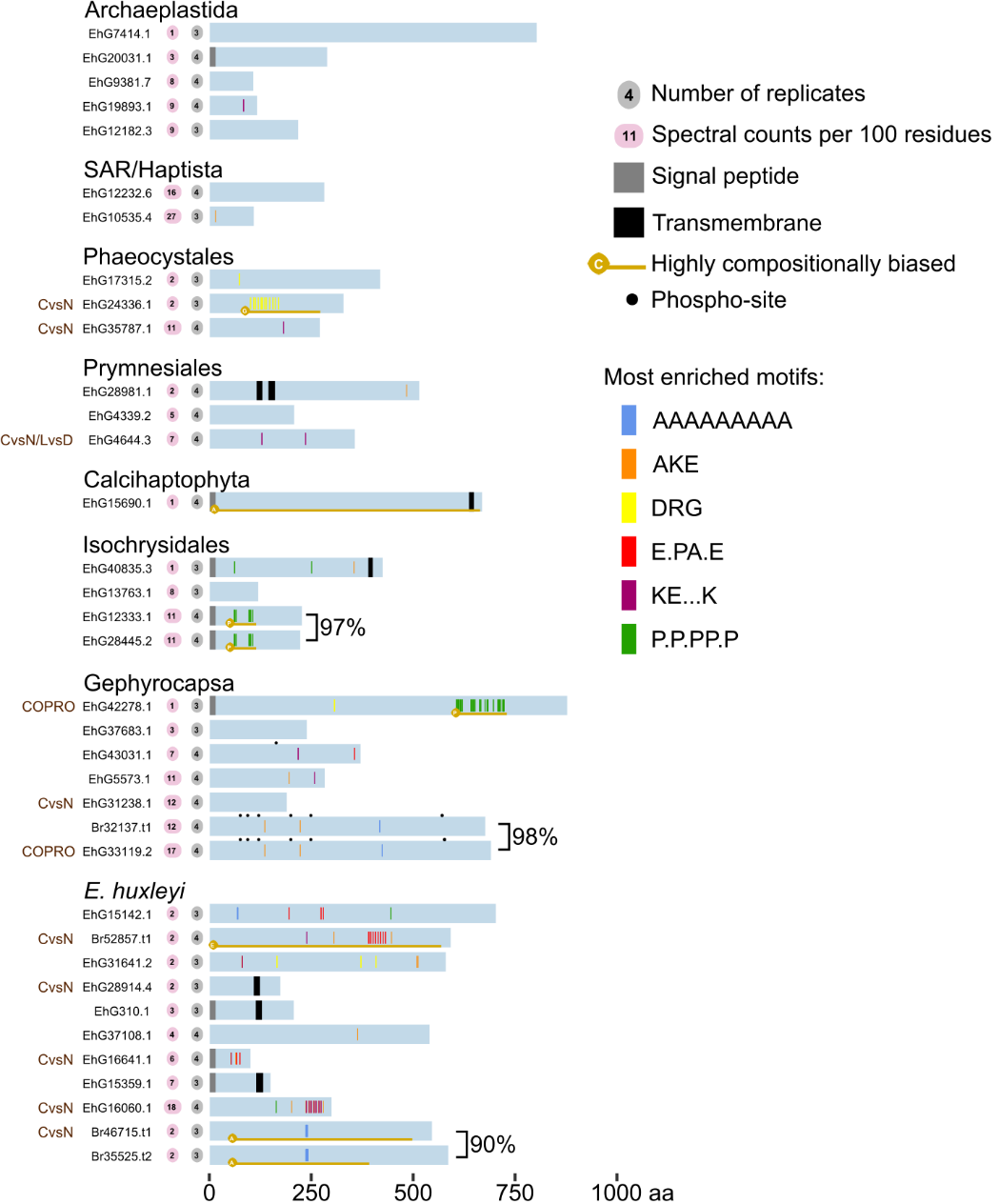


**Supplementary Figure 15: Coccosphere proteins without known domains.**


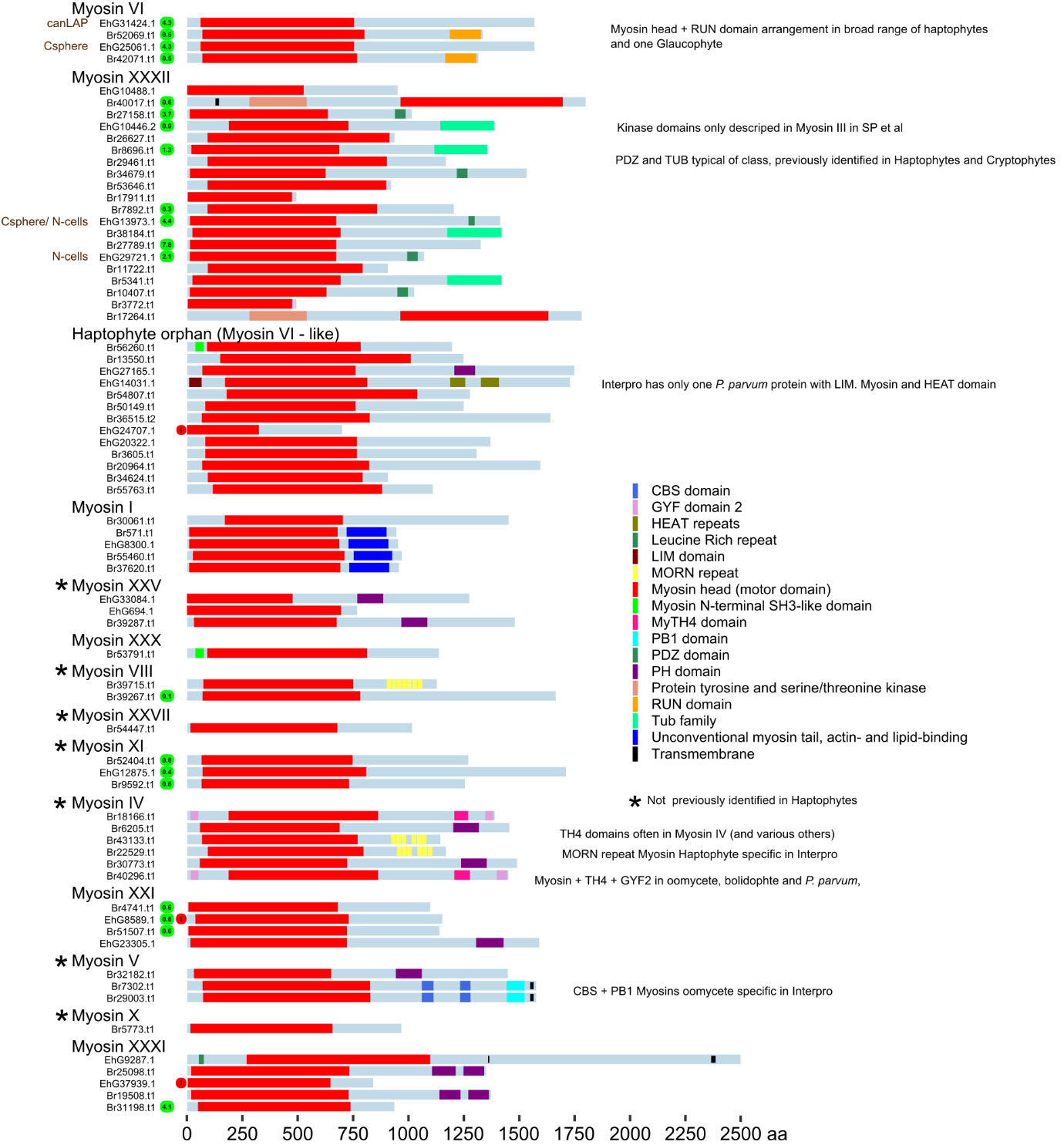


**Supplementary Figure 16: Schematic of all myosin sequences found in the Emihu2 proteome.** Myosins were identified in the Emihu2 proteome using the “Myosin_head” hidden Markov model associated with pfam domain PF00063 using the hmmsearch program from HMMER 3.3.2^6^. In line with the classification scheme developed by Sebé-Pedrós and co-workers^7^, only the myosin head domain was used for classification of the sequences. The Emihu2 myosins were compared to the database of myosins from Sebé-Pedrós *et al.* (2014) using phmmer and the best hit was used to assign the myosin class. Those myosins found in our calcification-related proteomics datasets are labelled to the left of the protein identifier. If a protein was identified in the whole-cell proteome data used for database quality control, the number of spectral counts per 100 residues is indicated with a lime green background. Classes of myosins that have not been previously identified in haptophytes^7^ are indicated with an asterisk. Incomplete gene models are indicated by an “I” on a red background.


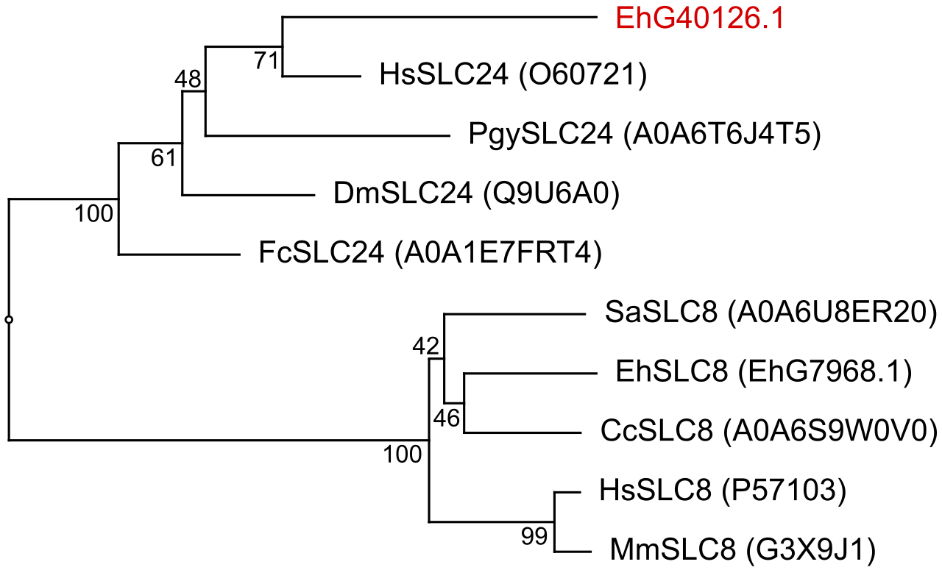
**Supplementary Figure 17: Phylogenetic analysis of SLC8 and SLC24 transporters of *E. huxleyi* and other model organisms, suggesting that EhG40126.1 is likely an SLC24-type exchanger.** The tree is based on a Mafft multiple sequence alignment (alignment strategy G-NIS-1) and was calculated using the neighbor-joining method with 1,000 bootstrap replicates. The other protein sequences for phylogenetic tree construction are: *Homo sapiens* (Hs), *Pavlova gyrans* (Pg), *Drosophila melanogaster* (Dm), *Fragilariopsis cylindrus* (Fc), *Scyphosphaera apsteinii* (Sa), *Chrysotila carterae* (Cc), and *Mus musculus* (Mm). In brackets: Uniprot [https://www.uniprot.org/] accession number of the protein.


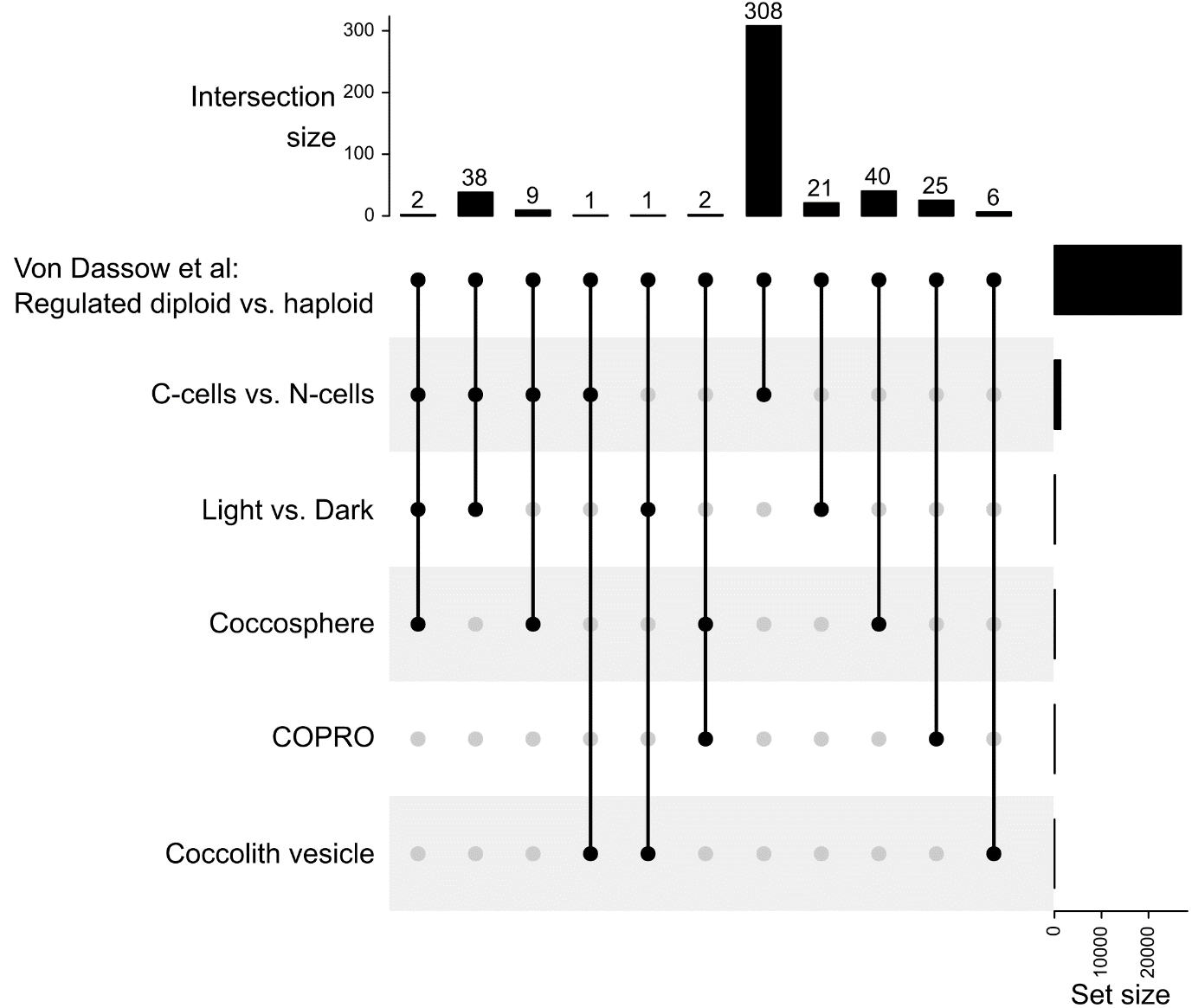
**Supplementary Figure 18**: Overlap between proteomics data sets from this paper and genes found to be differentially regulated in diploid C-cells vs non-calcifying haploid cells by von Dassow and co-workers ^8,9^. The raw data for the EST consensus sequences compared to the Emihu2 transcriptome using blastn are from published works ^8,9^. Hits with an e-value of 1E‑190 or smaller were accepted as putatively representing the same transcript.


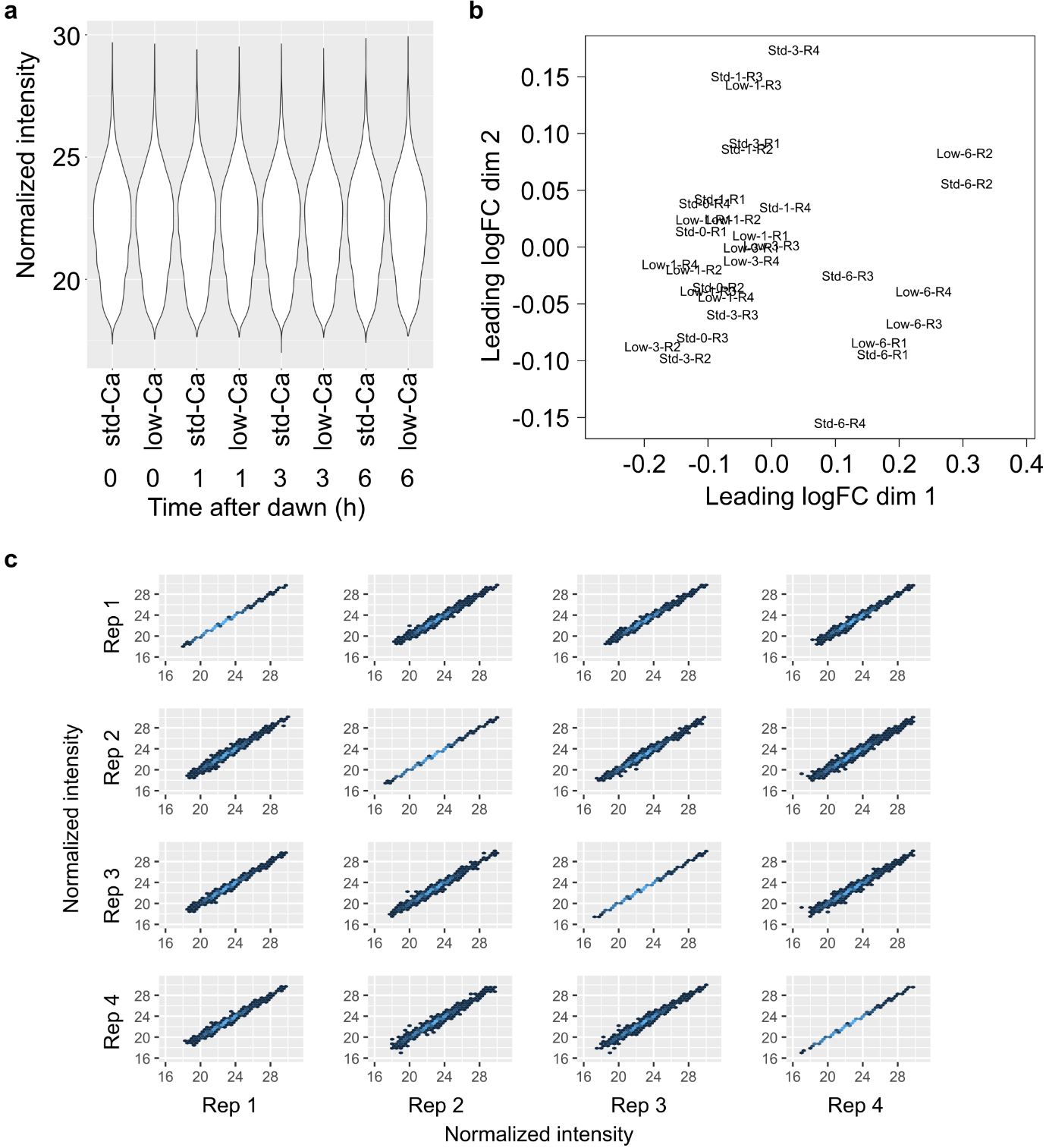


**Supplementary Figure 19: Plots for quality control of raw quantitative proteomics data and normalization methods for the recalcification data set.** (**a**) Violin plots of normalized intensity at the peptide level for each Ca / Time combination (three replicates per violin plot). (**b**) Multidimensional scaling plot using the log(2) fold change values for the 500 proteins displaying most change. Points are labelled as follows: ‘Low’ = low-Ca, ‘Std’ = std-Ca, the subsequent number indicates time point of sampling, and the final part indicates the replicate number. (**c**) Correlation plots of normalized intensity at the protein level for each pair of replicates. A lighter blue colour indicates more data at that point on the graph. R^2^ values are 1.00 for all same replicate comparisons, and otherwise are 0.99.


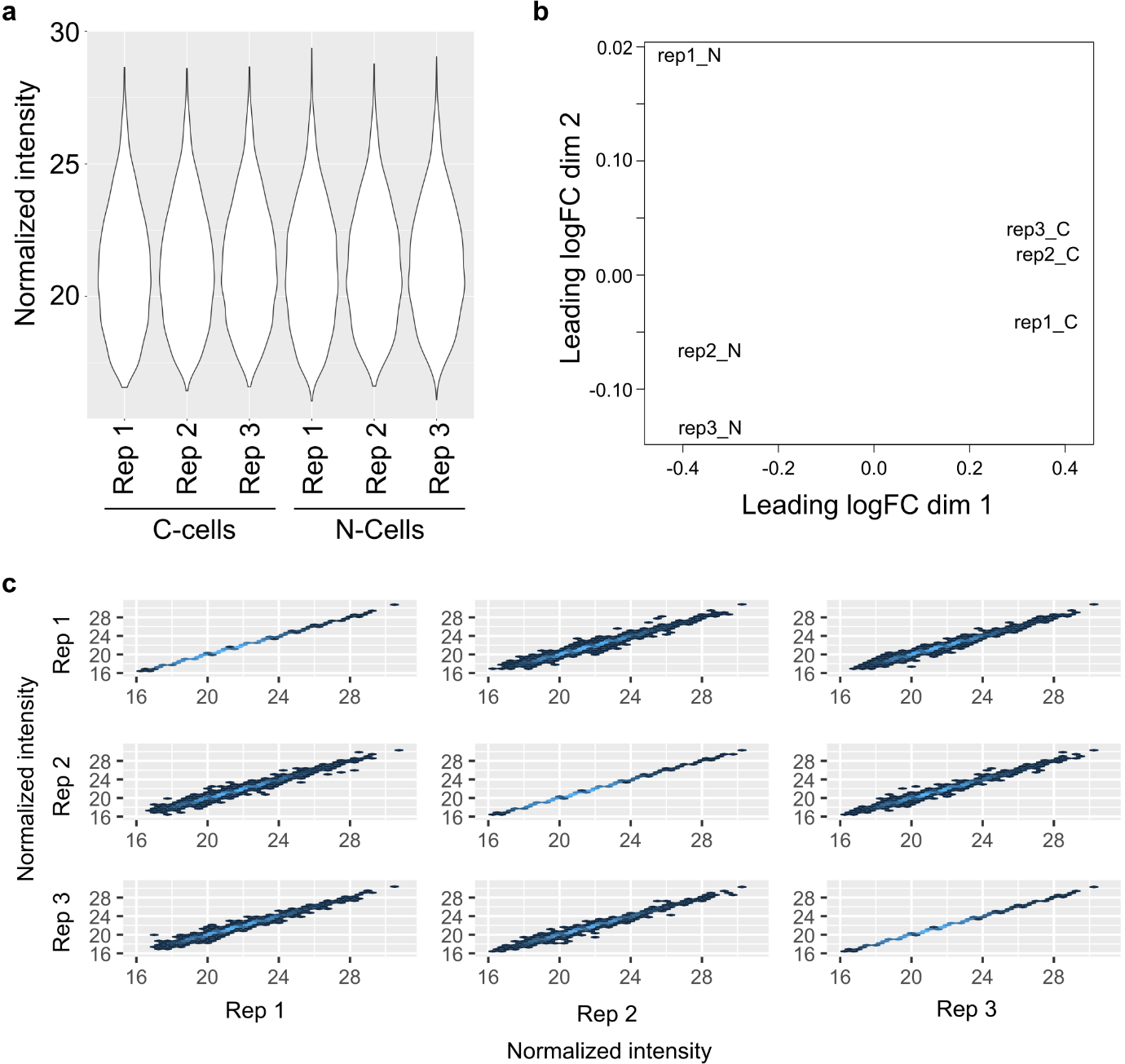


**Supplementary Figure 20: Plots for quality control of raw quantitative proteomics data and normalization methods for the N-cell vs. C-cell data set.** (**a**) Violin plots of normalized intensity at the peptide level for each replicate and condition. (**b)** Multidimensional scaling plot using the log(2) fold change values for the 500 proteins displaying most change. Points are labelled by replicate and condition. (**c**) Correlation plots of normalized intensity at the protein level for each pair of replicates. A lighter blue colour indicates more data at that point on the graph. R^2^ values are 1.00 for all same replicate comparisons, and otherwise are 0.99.

# **Supplementary Tables**

**Supplementary Table 1: Detailed statistics of haploid and diploid Emihu2 and Emihu1 genome assemblies.**

|  | **Emihu1** | **Emihu2 diploid** | **Emihu2**  **haploid** |
| --- | --- | --- | --- |
| **Number of scaffolds** | 7809 | 600 | 165 |
| **Total size of scaffolds** | 1.68E+08 | 1.96E+08 | 97,876,491 |
| **Longest scaffold** | 3,018,814 | 3,529,537 | 3,529,537 |
| **Shortest scaffold** | 1,000 | 2,265 | 2,265 |
| **Number of scaffolds > 1K nt** | 7,800 | 600 | 165 |
| **Number of scaffolds > 10K nt** | 1,165 | 586 | 162 |
| **Number of scaffolds > 100K nt** | 323 | 384 | 139 |
| **Number of scaffolds > 1M nt** | 23 | 45 | 36 |
| **Number of scaffolds > 10M nt** | 0 | 0 | 0 |
| **N50** | 404,808 | 682,851 | 1,120,510 |
| **L50** | 109 | 84 | 31 |
| **NG50** | 293,447 | 657,487 | 1,069,152 |
| **LG50** | 157 | 88 | 32 |
| **%A** | 15.97 | 16.81 | 16.83 |
| **%C** | 30.51 | 33.21 | 33.16 |
| **%G** | 30.57 | 33.17 | 33.19 |
| **%T** | 15.95 | 16.80 | 16.80 |
| **Total Number of Ns** | 11,700,982 | 0 | 0 |
| **%N** | 6.97 | 0 | 0 |
| **Repeat regions (%)** | 34.05 | 35.82 | - |
| **Unclassified repeats (%)** | 26.84 | 27.27 | - |

**Supplementary Table 2: BUSCOs called as missing in the Emihu2 proteome, which in fact have putative orthologs in the Emihu2 genome.**

| **OrthoDB ID** | **Annotation** | **Best**  **blastp hit** | **Blastp**  **e-value** | **Best**  **tblastn hit** | **Start position** | **tblastn**  **e-value** |
| --- | --- | --- | --- | --- | --- | --- |
| 1107630at2759 | Dolichyl-phosphate beta-glucosyltransferase | EhG32341.1 | 1.00E-27 | 000041F_arrow | 446700 | 6.00E-24 |
| 1312453at2759 | WD40-repeat-containing domain | Br1416.t1 | 1.00E-50 | 000104F_arrow | 567380 | 4.00E-35 |
| 1364586at2759 | rRNA-processing protein Fcf1/Utp23 | EhG25184.1 | 4.00E-16 | 000000F_arrow | 75601 | 3.00E-27 |
| 1428854at2759 | RNA recognition motif domain | Br51559.t1 | 3.00E-88 | No tblastn hit | NA | NA |
| 1445102at2759 | Glycosyltransferase 2-like | EhUM10193  EhG6702.1 | 2.00E-26  1.00E-23 | 000015F_arrow  No tblastn hit | 1281754  NA | 1.00E-21  NA |
| 271586at2759 | Tetratricopeptide repeat | Br13955.t1 | 2.00E-22 | 000014F_arrow | 433799 | 1.00E-21 |
| 388820at2759 | WD40 repeat | Br16950.t1 | 1.00E-64 | 000060F_003_arrow | 362457 | 2.00E-17 |
| 39650at2759 | DNA polymerase epsilon catalytic subunit | Br34051.t1 | 0.00E+00 | 000040F_arrow | 385707 | 7.00E-28 |
| 457861at2759 | Autophagy protein 5 | EhG3449.2 | 3.00E-12 | 000095F_arrow | 66465 | 1.00E-11 |
| 464990at2759 | Eukaryotic translation initiation factor 2A | Br11131.t1 | 5.00E-40 | 000025F_004_arrow | 179680 | 2.00E-18 |
| 598949at2759 | AP-3 complex subunit delta | EhG122.1 | 7.00E-37 | 000135F_arrow | 79810 | 2.00E-19 |
| 687505at2759 | DnaJ domain | EhG30819.1 | 1.00E-49 | 000079F_arrow | 563675 | 9.00E-16 |

**Supplementary Table 3: EukProt proteomes used in the phylostratigraphic analysis**. Note, for the haptophytes, the prefix is order level: Pav = Pavlovales; Pry = Prymnesiales; Coc = Coccolithales; Iso = Isochrysidales; Zyg = Zygodiscales; Pha = Phaeocystales.

|  | **EukProt ID** | **Code in analysis** | **Species name** |
| --- | --- | --- | --- |
| **1** | EP00168 | Rho_PorphyraPur | *Porphyra purpurea* |
| **2** | EP00185 | Rho_PorphyriPur | *Porphyridium purpureum* |
| **3** | EP00198 | Chl_Crei | *Chlamydomonas reinhardtii* |
| **4** | EP00260 | Chl_Atha | *Arabidopsis thaliana* |
| **5** | EP00274 | Gla_Cglo | *Cyanoptyche gloeocystis* |
| **6** | EP00276 | Gla_Gwit | *Gloeochaete wittrockiana* |
| **7** | EP00279 | Cry_Gthe | *Guillardia theta* |
| **8** | EP00282 | Cry_Hand | *Hemiselmis andersenii* |
| **9** | EP00296 | Kat_Rtru | *Roombia truncata* |
| **10** | EP00297 | Pal_Pbil | *Palpitomonas bilix* |
| **11** | EP00298 | Cen_Acan | *Acanthocystis* sp HF-20 |
| **12** | EP00299 | Cen_Pcan | *Pterocystis canadensis* |
| **13** | EP00300 | Cen_Cho | *Choanocystis* sp HF-7 |
| **14** | EP00303 | Pav_Egay | *Exanthemachrysis gayraliae* RCC1523 |
| **15** | EP00304 | Pav_Pgyr | *Pavlova gyrans* CCMP608 |
| **16** | EP00305 | Pav_Dlut | *Diacronema lutheri* RCC1537 (*Pavlova lutheri*) |
| **17** | EP00307 | Pav_Reb | *Rebecca salina* RCC1486 |
| **18** | EP00308 | Coc_Clep | *Calcidiscus leptoporus* |
| **19** | EP00309 | Coc_Ccar | *Chrysotila carterae* |
| **20** | EP00310 | Coc_Cbra | *Coccolithus braarudii* |
| **21** | EP00311 | Iso_Igal | *Isochrysis galbana* |
| **22** | EP00312 | Iso_Isp1244 | *Isochrysis* sp CCMP1244 |
| **23** | EP00313 | Iso_Isp1324 | *Isochrysis* sp CCMP1324 |
| **24** | EP00315 | Iso_Goc | *Gephyrocapsa oceanica* |
| **25** | EP00316 | Zyg_Sap | *Scyphosphaera apsteinii* |
| **26** | EP00317 | Pha_Pant | *Phaeocystis antarctica* |
| **27** | EP00323 | Pry_Ctob | *Chrysochromulina tobin* |
| **28** | EP00324 | Pry_Drot | *Dicrateria rotunda* |
| **29** | EP00325 | Pry_Hbre | *Haptolina brevifila* |
| **30** | EP00327 | Pry_Ppar | *Prymnesium parvum* |
| **31** | EP00328 | Pry_Ppol | *Prymnesium polylepis* |
| **32** | EP00339 | Alv_Tfus | *Tiarina fusus* |
| **33** | EP00346 | Alv_Otri | *Oxytricha trifallax* |
| **34** | EP00439 | Alv_Sym | *Symbiodinium* sp Y106 |
| **35** | EP00466 | Rhi_Bnat | *Bigelowiella natans* |
| **36** | EP00530 | Str_Ptri | *Phaeodactylum tricornutum* |
| **37** | EP00583 | Str_Tpse | *Thalassiosira pseudonana* |

**Supplementary Table 4: HSP90-like proteins with transcript level support in the Emihu2 proteome.** Signal peptide prediction was performed with SignalP v5.0 ^10^.

| **ID** | **Signal peptide** |
| --- | --- |
| EhG570.1 | No |
| EhG1535.3 | Yes |
| EhG7640.1 | No |
| EhG8172.6 | No |
| EhG9823.3 | No |
| EhG18090.2 | No |
| EhG23268.2 | Yes |
| EhG29690.4 | No |
| EhG31490.1 | No |
| EhG35024.1 | Yes |

**Supplementary Table 5: Proteins of interest identified due to overlap between data sets.** The ‘Category’ field describes the putative intracellular role of the protein.

| **Overlap in datasets** | **ID** | **Category** | **OG age** | **Notes** |
| --- | --- | --- | --- | --- |
| COPRO, coccosphere | EhG33272.1 | CV membrane protein | *E. huxleyi* age | MAM domain (extracellular adhesive domain) containing protein |
|  | EhG42278.1 | CV lumen protein | *Gephyrocapsa* age | Signal peptide, P rich regions, with many P.P.PP.P repeats |
| COPRO, CvsN | EhG40669.2 | CV lumen protein | *Gephyrocapsa* age | Signal peptide. Pentapeptide repeat containing. Downregulated in C-cells vs N-cells (0.56x). |
| COPRO, CvsN, LvsD | EhG6475.1 | CV lumen protein | Archaeplastida age or older | Signal peptide |
| CV, Coccosphere | EhG30140.1 | Cytoplasmic, CV-associated protein | Archaeplastida age or older | Actin. Found in only 2 replicates in the coccosphere Std-Ca data, so not included in the main coccosphere results table. |
| CV, LvsD | EhG12212.4 | Cytoplasmic, CV-associated protein | Phaeocystales age | 1.3x up in Light vs Dark dataset. FOP domain containing. Potentially involved in microtubule dynamics. |
| CV, CvsN | EhG3936.1 | CV membrane protein | Archaeplastida age or older | ATP synthase a/b subunit. Downregulated (0.52x) in C-cells relative to N cells. |
|  | EhG16179.1 | Cytoplasmic, CV-associated protein | Phaeocystales age | 1.4x upregulated in C-cells vs N-cells. |
| Coccosphere, CvsN, LvsD | EhG4644.3 | Coccosphere protein | Prymnesiales age | Down regulated in C-cells, upregulated in Light vs Dark dataset (1.4x). Two KE…K motifs, which is enriched in a number of coccosphere proteins. |
|  | EhG9529.10 | Plasma membrane protein | Archaeplastida age or older | Bicarbonate transporter. Down regulated in C-cells (0.5x) and downregulated in Light vs Dark dataset (0.7x). |
|  | EhG31109.3 | Coccosphere protein | Archaeplastida age or older | Beta carbonic anhydrase. Downregulated in C-cells (0.8x), downregulated in the day (0.8x) |
| Coccosphere, CvsN | EhG6615.6 | Coccosphere protein | Archaeplastida or older | Adenylate-forming enzyme |
|  | EhG31129.1 | Coccosphere protein | *E. huxleyi* age | Signal peptide |
|  | EhG13973.1 | Cytoplasmic, CV-associated protein | Archaeplastida or older | Myosin |
|  | EhG16060.1 | Coccosphere protein | *E. huxleyi* age | Downregulated in C-cells (0.6x) |
|  | EhG16641.1 | Coccosphere protein | *E. huxleyi* age | Signal peptide, E containing motifs |
|  | EhG21927.2 | Coccosphere protein | *E. huxleyi* age | ShK domain-like |
|  | EhG22535.1 | Coccosphere protein | Archaeplastida or older | PH domain containing protein. May act in phosphatidyl-inositol binding and membrane recruitment of proteins. Downregulated in C-cells (0.9x). |
|  | EhG24336.1 | Coccosphere protein | Phaeocystales age | Upregulated in C-cells (1.2x). |
|  | EhG28914.4 | Plasma membrane protein | *E. huxleyi* age | Single transmembrane span |
|  | EhG31238.1 | Coccosphere protein | Gephyrocapsa age |  |
|  | EhG35787.1 | Coccosphere protein | Phaeocystales age | A single KE…K motif, which is enriched in a number of coccosphere proteins. |
|  | EhG35934.1 | Coccosphere protein | *E. huxleyi* age | NUDIX domain (Found in pyrophosphohydrolases). |
|  | EhG37998.2 | Coccosphere protein | Archaeplastida or older | Chaperonin 10kDa subunit |
|  | EhG13464.1 | Coccosphere protein | Archaeplastida or older | Aminotransferase class V |
|  | EhG8541.1 | Coccosphere protein | Archaeplastida or older | Tyrosinase domain and peptidoglycan binding domain |
|  | Br52857.t1 | Coccosphere protein | *E. huxleyi* age | E rich protein, with E.PA.E motifs. Upregulated in C-cells (1.2x). |

**Supplementary Table 6: Flagella genes found in the Emihu1 and Emihu2 proteomes.**

| **Type of flagella gene** | **Total number searched for by von Dassow *et al.*** ^8^ | **Missing in Emihu1** | **Missing in Emihu2** |
| --- | --- | --- | --- |
| Outer dynein arm | 19 | 3 | 0 |
| Inner dynein arm | 17 | 2 | 0 |
| Central pair | 4 | 0 | 0 |
| Radial spoke associated proteins | 5 | 2 | 1 |
| Intraflagellar transport | 11 | 5 | 4 |
| Miscellaneous | 7 | 0 | 0 |
| Eukaryotic flagella and basal-body | 19 | 7 | 4 |
| **Total:** | **82** | **19** | **9** |
|  |  |  |  |
|  | Missing [%] | 23.2 | 11.0 |

**Supplementary Table 7: Availability of proteomics data.**

| **Project Name** | **Accession** | **Link** |
| --- | --- | --- |
| Coccolith proteins from *E. huxleyi* (COPROs) | PXD027059 | https://www.ebi.ac.uk/pride/archive/projects/PXD027059 |
| *E. huxleyi* coccosphere-associated proteins identification | PXD027440 | https://www.ebi.ac.uk/pride/archive/projects/PXD027440 |
| Coccolith vesicle proteins from *E. huxleyi* | PXD027481 | https://www.ebi.ac.uk/pride/archive/projects/PXD027481 |
| *E. huxleyi* proteomic database quality control | PXD027501 | https://www.ebi.ac.uk/pride/archive/projects/PXD027501 |
| Quantitative changes in the *E. huxleyi* proteome during recalcification | PXD027515 | https://www.ebi.ac.uk/pride/archive/projects/PXD027515 |
| Quantitative comparison of *E. huxleyi* proteome in C-cells, N-cells, and in light and dark phase | PXD027567 | https://www.ebi.ac.uk/pride/archive/projects/PXD027567 |

# **Supplementary Notes**

## **Supplementary Note 1: Further details of sequencing and assembly methods**

In total 8.86 Gb of data were generated on two PacBio Sequel SMRT cells for the generation of the new genome (Supplementary Table 8, Supplementary Figure 21a,b)

**Supplementary Table 8: Summary of RAW PacBio data used for genome assembly.**

|  | 1^st^ SMRT cell | | 2^nd^ SMRT cell | |
| --- | --- | --- | --- | --- |
| # reads | 593,236 | | 335,155 | |
| P1 | 0.57 | | 0.32 | |
|  | Total reads | CCS reads | Total reads | CCS reads |
| Output [Gb] | 5.66 | 4.84 | 3.2 | 2.68 |
| max. length [kb] | 242 | 168 | 247 | 167 |

**Genome assembly**

In SMRTLink v7.0.1 the default json parameter file was adapted as follows: falcon_ns.task_options.HGAP_SeedCoverage_str 30, falcon_ns.task_options.HGAP_ GenomeLength _str 170000000, falcon_ns2.task_options.save_las_for_unzip true. The SMRTLink data file was converted to a Falcon-compatible file using the “hgap4_adapt” script, which comes with the Anaconda installation of Falcon unzip ^11^ was used to generate an initial phased assembly. Purge Haplotigs ^12^ was used to improve the haploid assembly as follows. After conversion of the subreads BAM file to fasta format (using bam2fasta from the SMRTLink repository), all genomic subreads were mapped back against the draft genome using minimap2 (parameter: -t 20, -ax map-pb, -secondary=no). This made the input for “purge_haplotigs hist”, generating a histogram (Supplementary Figure 21c) that was used as detailed in the Purge Haplotigs manual to define cut-off parameters for the subsequent “purge_haplotigs cov” command as: -l 10 -m 70 -h 190. The final step of the Purge Haplotigs pipeline uses an alignment score cut-off (parameter “-a”) to determine if aligned contigs should be called as haplotigs or not. From the absence of a double peak in the read-mapping step (Supplementary Figure 21c), we deduced that nearly the entire genome assembly was diploid (i.e. sufficient heterozygosity was present that homologous genomic regions were not collapsed into single contigs during assembly). In turn, this means that we expect the genomic sequence to be approximately equal distributed between primary contigs and haplotigs. To achieve this, we optimised the final step of Purge Haplotigs by repeatedly running the analysis while varying “-a” from 0 to 100 (Supplementary Figure 21d). We also recorded the proportion of high quality Isoseq reads mapping back to the resultant primary contigs for each value of “-a” (Supplementary Figure 21d). A final parameter value of 37 was chosen, since this is the point at which genomic sequence length was split equally between primary contigs and haplotigs, and the value at while mapping of Isoseq reads back to the primary contigs plateaued close to 100%, suggesting good completeness with respect to coding sequence.

The final genome (Emihu2) was considerably more contiguous than the Emihu1 genome (Supplementary Figure 21e, Supplementary Figure 22).


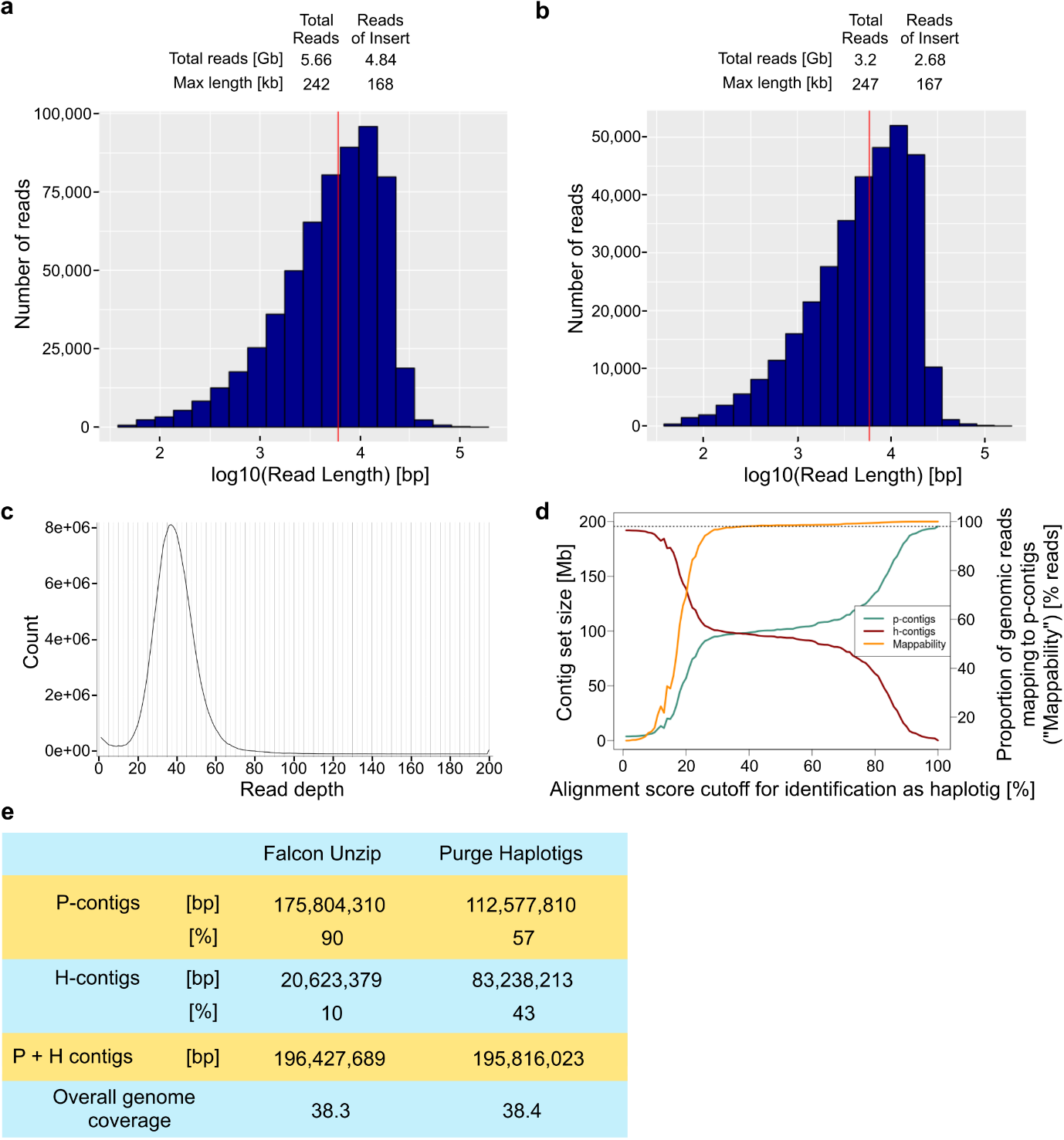
**Supplementary Figure 21: Assembly of the genome.** The data output for each of the two SMRT cells is described in (**a**) and (**b**). (**c**) The mapping of the genomic subreads back against the Falcon unzip assembly (primary contigs and haplotigs). (**d**) The effect of varying the -a parameter (alignment score cutoff) on the amount of DNA assigned to primary contigs (p-contigs) or haplotigs (h-contigs) by the Purge Haplotigs pipeline. In addition, the proportion of the high quality Isoseq reads that map to the p-contigs for given values of ‘a’ is shown. (**e**) Distribution of genomic sequence between p-contigs and h-contigs after the Falcon unzip step, and after the Purge Haplotigs pipeline.


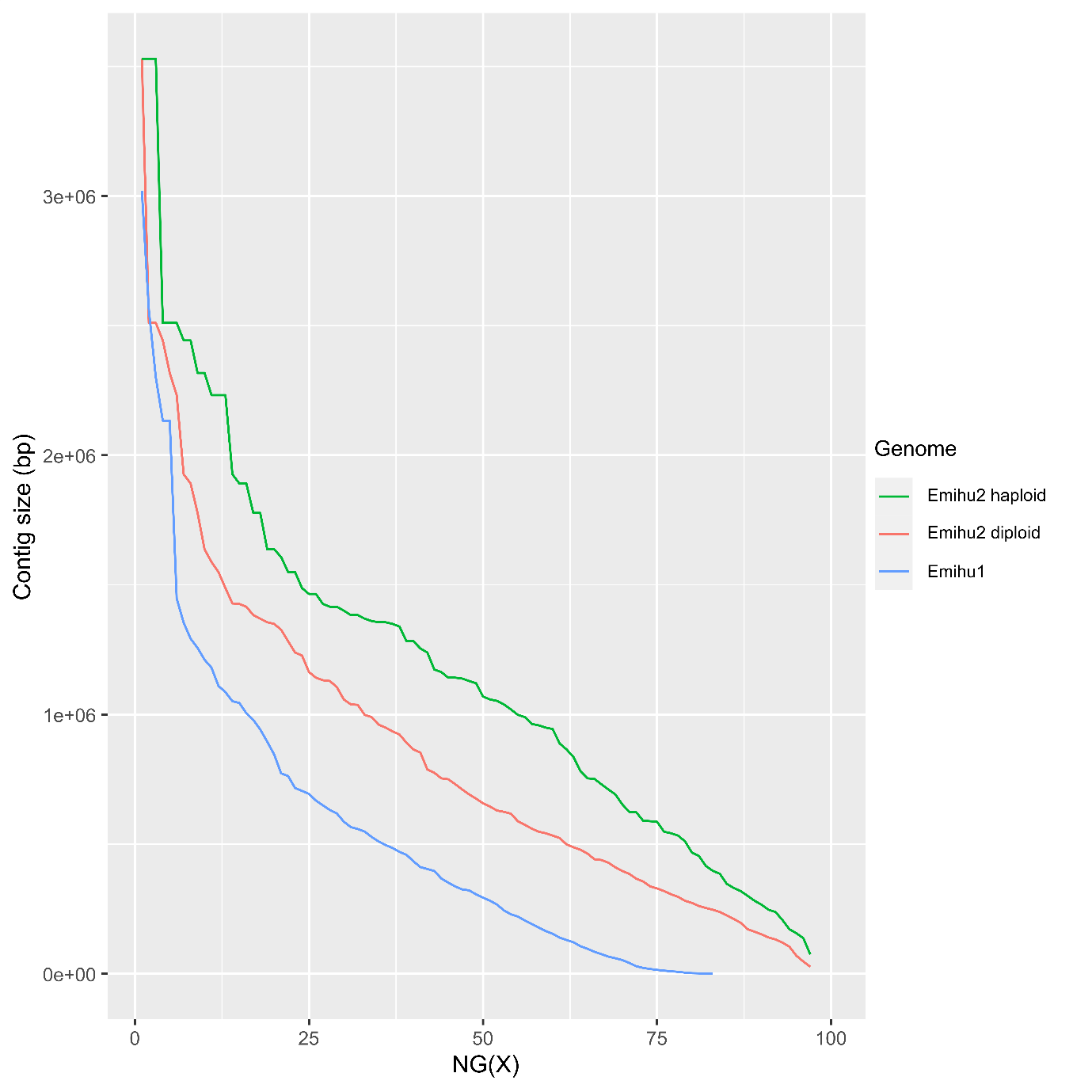


**Supplementary Figure 22: Comparison of contiguity in the Emihu1 and the haploid and diploid versions of the Emihu2 genome.**

**Repeat content**

There was little difference between the new and JGI genomes in terms of repeat content (Supplementary Figure 23, Supplementary Table 9). Both contained between 30 and 40% repeats, the new genome having a little more, as would be expected for a genome based on long-read sequencing. The dominant repeats in the genome are unclassified.


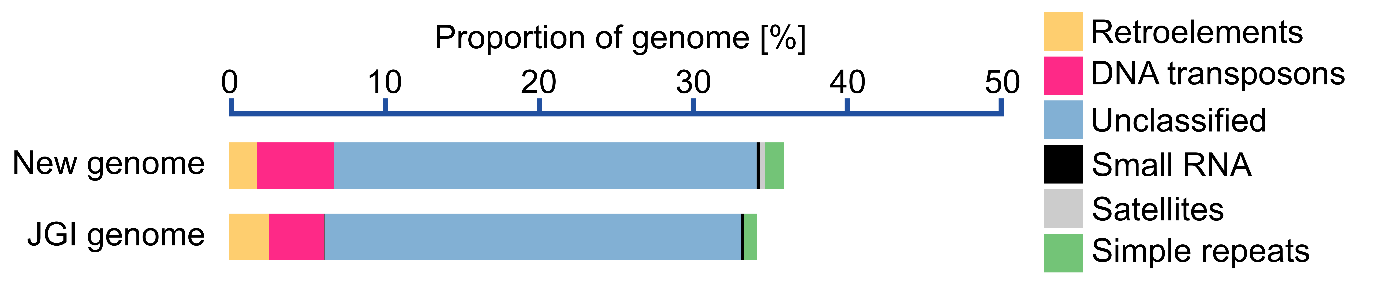


**Supplementary Figure 23: Repeat content of the diploid Emihu2 genome in comparison to the Emihu1 genome.** Repeats and transposable elements were identified by a combination of homology-based and *de novo* approaches using Repeatmasker v.4.1.2-p1 ^13^. A *de novo* repeat library was generated using RepeatModeler v2.0.1 with enabled LTR discovery pipeline and combined with the *Emiliania* repeat entries of the Repbase v23.08 library ^14^. Annotation of repeats was based on the libraries RepBase v23.08 and Dfam 3.3 using RMBlast.

**Supplementary Table 9: Main types of predicted repeats in *E. huxleyi* diploid genome.**

|  | **Emihu2 genome** | | **Emihu1 genome** | |
| --- | --- | --- | --- | --- |
|  | Total length (bp) | Proportion of sequence (%) | Total length (bp) | Proportion of sequence (%) |
| **Retroelements** | 3,576,302 | 1.826358 | 4,369,133 | 2.604907 |
| SINEs: | 0 | 0 | 0 | 0 |
| Penelope | 61,896 | 0.031609 | 39,280 | 0.023419 |
| LINEs: | 731,298 | 0.373462 | 470,316 | 0.280406 |
| CRE/SLACS | 46,801 | 0.0239 | 115,487 | 0.068854 |
| L2/CR1/Rex | 34,587 | 0.017663 | 8403 | 0.00501 |
| R1/LOA/Jockey | 345,939 | 0.176665 | 20,847 | 0.012429 |
| R2/R4/NeSL | 18,235 | 0.009312 | 18,752 | 0.01118 |
| RTE/Bov-B | 169,129 | 0.086371 | 214,727 | 0.128022 |
| L1/CIN4 | 22,180 | 0.011327 | 0 | 0 |
| LTR elements | 2,845,004 | 1.452896 | 3,898,817 | 2.324501 |
| BEL/Pao | 194,938 | 0.099552 | 310,133 | 0.184903 |
| Ty1/Copia | 1,068,481 | 0.545656 | 1,029,301 | 0.613676 |
| Gypsy/DIRS1 | 1,083,985 | 0.553573 | 1,297,775 | 0.773742 |
| Retroviral | 0 | 0 | 0 | 0 |
| **DNA transposons** | 9,676,152 | 4.941451 | 5,965,197 | 3.556491 |
| hobo-Activator | 1,833,357 | 0.936265 | 1,595,452 | 0.951219 |
| Tc1-IS630-Pogo | 255,330 | 0.130393 | 74,975 | 0.044701 |
| En-Spm | 0 | 0 | 0 | 0 |
| MuDR-IS905 | 0 | 0 | 0 | 0 |
| PiggyBac | 173,064 | 0.088381 | 271,698 | 0.161988 |
| Tourist/Harbinger | 37,400 | 0.0191 | 25,021 | 0.014918 |
| Other (Mirage, P-element, Transib) | 0 | 0 | 0 | 0 |
| **Rolling-circles** | 0 | 0 | 87820 | 0.052359 |
| **Unclassified:** | 53,393,654 | 27.26725 | 45,010,496 | 26.83556 |
| **Total interspersed repeats** | **66,646,108** | **34.03506** | **55,344,826** | **32.99696** |
|  |  |  |  |  |
| **Other repeats:** |  |  |  |  |
| Small RNA: | 432,354 | 0.220796 | 325,986 | 0.194355 |
| Satellites: | 649,431 | 0.331654 | 0 | 0 |
| Simple repeats: | 2,422,873 | 1.237321 | 1,352,525 | 0.806385 |
| Low complexity: | 0 | 0 | 0 | 0 |
| **Total repetitive DNA** | **70,150,766** | **35.82** | **57,111,157** | **34.05** |

**RNA extraction, library preparation and PacBio IsoSeq sequencing.**

To extract RNA from the cell-covered filters using the Direct-zol RNA purification kit (Zymo Research), the Trizol reagent was first added to the filters and the cells were washed by pipetting up and down from the filter. To help break open the cells, a scoop of autoclaved glass beads (diameter: 0.25 – 0.5 mm) was added and the samples were vortexed for 25 sec. Otherwise the protocol from the kit was followed.

In addition to sequencing on the Sequel, an initial RNA sample, extracted from cells during the light-phase, was also sequenced on the RSII system and these data were included in the data used to build the new transcriptome (Sample ‘RSII data’ in Supplementary Table 10). For RSII sequencing, sample preparation was according to the protocol "Isoform Sequencing (Iso-Seq™) Using the SageELF™ Size Selection System" of Pacific Biosciences and sequencing was with DNA polymerase version P4 and sequencing chemistry version C4 for 6 h on the RSII.

In total 331 Gb of data were collected to generate the new transcriptome, resulting in 621,030 high quality isoform sequences.

**Supplementary Table 10: Summary of PacBio Isoseq data used for constructing the *E. huxleyi* transcriptome.**

|  | **Light** | **Dark** | **Low Ca^2+^** | **P-starved** | **RSII data** | **sum** |
| --- | --- | --- | --- | --- | --- | --- |
| # Polymerase reads | 2,905,128 | 3,015,493 | 3,145,992 | 3,159,142 | 4,491,579 | 16,717,334 |
| Polymerase reads [Gb] | 58.2 | 61.5 | 59.3 | 65.9 | 86.5 | 331.3 |
| # CCS reads | 2,319,833 | 2,348,867 | 2,383,116 | 2,503,693 | 2,220,544 | 11,776,053 |
| CCS reads [Gb] | 1.9 | 2.0 | 2.0 | 2.1 | 4.5 | 12.4 |
| # FLNC reads | 1,569,484 | 1,552,821 | 1,500,402 | 1,650,722 | 1,714,413 | 7,987,842 |
| FLNC reads [Gb] | 1.0 | 1.1 | 1.0 | 1.1 | 2.9 | 7.1 |
| # LQ isoforms | 5,572 | 5,993 | 6,581 | 6,233 | 8,554 | 32,933 |
| LQ isoforms [Mb] | 3.1 | 3.1 | 3.5 | 3.3 | 16.4 | 29.5 |
| # HQ isoforms | 113,192 | 133,067 | 121,676 | 139,452 | 113,643 | 621,030 |
| HQ isoforms [Mb] | 78.3 | 97.9 | 85.9 | 102.0 | 176.7 | 540.9 |

## **Supplementary Note 2: ORF selection and making the predicted proteome**

**Defining ORFs and proteome generation.**

ORF selection was carried out using the script ‘FinalORFs.R’ (Figshare: doi.org/10.6084/m9.figshare.20464293.v1). The first step in the procedure to select ORFs looks for clear evidence as to whether the sequence derived from the genome (GenSeq) or the isoseq reads (IsoSeq) should be preferred. Only considering long IsoSeq reads that are the most similar to the genomic sequence effectively selects against IsoSeq sequences with sequencing errors. For the majority of gene models, the genomic sequence was used for ORF prediction, but for just over 20% the IsoSeq sequence was used (Supplementary Figure 24). In the second step, an ORF is chosen from the ORFs generated by the three prediction tools. This decision tree prioritises ORFs with more pfam domains, and then prioritising ORFs that are annotated as complete by TransDecoder or gmst, and then ORFs that are longer than the alternatives (Supplementary Figure 24). If the chosen ORF was annotated as incomplete the getorf ORF was considered, and chosen if it was of similar length and had the same number of, or more, pfam domains. This seemed sensible because, for our data, the gmst and TransDecoder start site selection models often seem to discount a methionine close to the end of, and in the same frame as, the ‘incomplete’ 5’ end of the transcript. Given our very limited knowledge of the molecular biology of gene expression in haptophytes, it seemed reasonable to assume that in these instances the start site selection models used by gmst and TransDecoder were not appropriate.

In addition, ORFs from unmapped reads were added to the predicted proteome we used as a database for proteomics (to avoid false *E. huxleyi* protein identifications due to prokaryote derived spectra matching an *E. huxleyi* protein). These sequences can be identified in the proteome fasta file by the prefix “EhUM”. These were selected by a similar procedure to that described above (see code on Figshare doi.org/10.6084/m9.figshare.20464293.v1). Redundancy was removed from the chosen set of ORFs for the unmapped reads using CD‑Hit^15^ (with ‘-c 1.0’). This resulted in 8,736 ORFs which were added to the database.


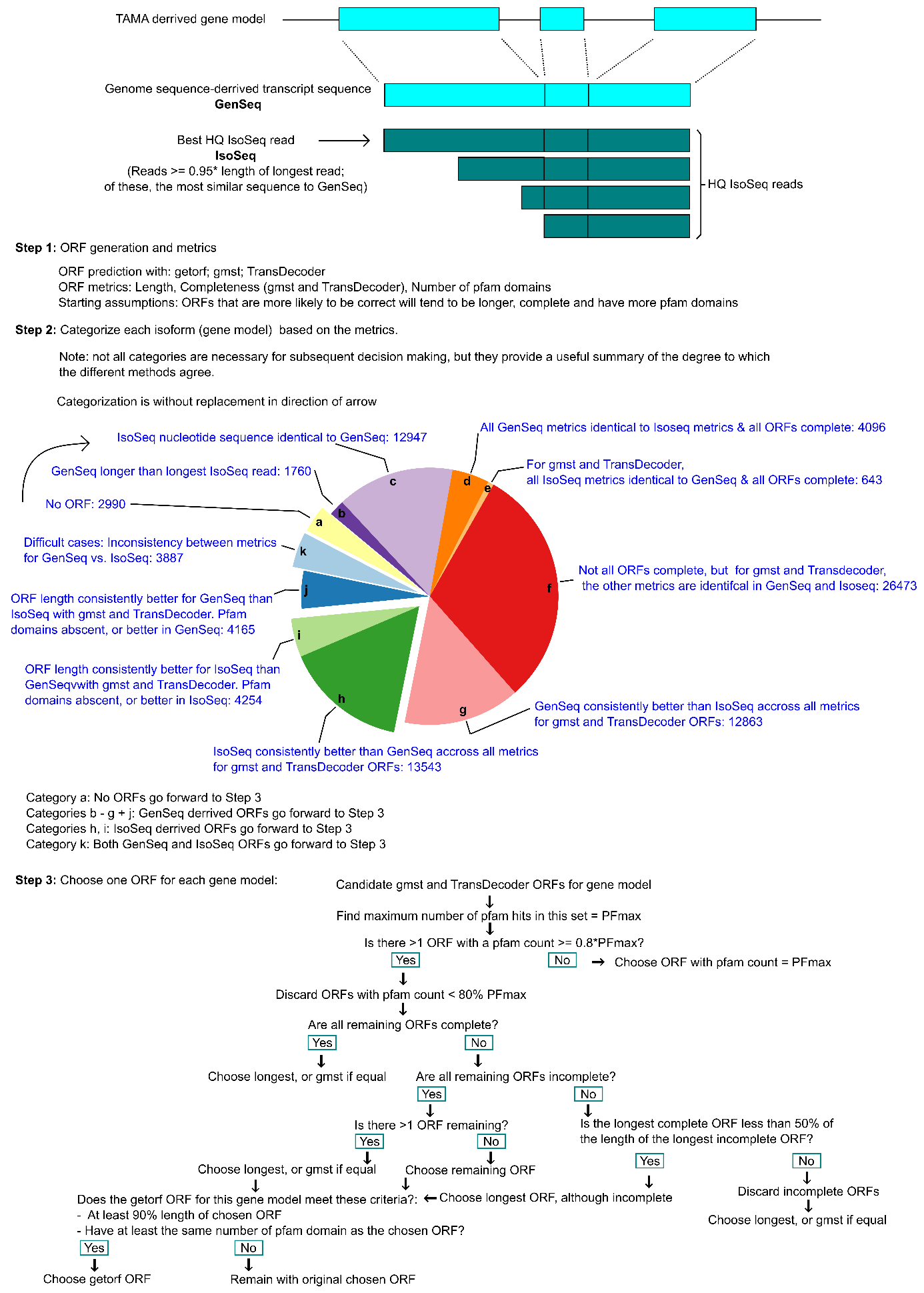


**Supplementary Figure 24: Graphical summary of the ORF selection procedure described in the text and implemented in the R code.**

## **Supplementary Note 3: Data sources for database quality control**

For whole cell extracts, cultures of *E. huxleyi* were grown to mid-log phase, harvested the volume required for 4x10^8^ cells by centrifugation for 10 min at 2,000g, and resuspended in 400 µl of extraction buffer (100 mM HEPES pH 7.5, 2 mM DTT, 75 mM NaCl, 0.5% (w/v) PVPP, 1x Sigma cOmplete protease inhibitor cocktail, with the addition of the following detergents, depending on the sample:

Sample 1: 1% SDS

Sample 2: 1% SDOC

Sample 3: 1% DDM

Sample 4: 1% IGEPAL

Samples were sonicated on ice four times at 30% duty with a pause of 30 s between each sonication, followed by centrifugation at 20,000g and 4˚C for 30 min. Sample clean-up and digests were with a FASP/tryptic digest procedure, as described in ‘Proteomic methods’. Coccolith associated proteins samples are the same as those described in ‘Proteomic methods’. However only the tryptic digests from the three biological replicates were used in this analysis. Arabidopsis data were downloaded from the PRIDE repository:

| **ID in Fig S4** | **Study refer-ence** | **Download link** |
| --- | --- | --- |
| 1 / 3 | 16 | ftp://ftp.pride.ebi.ac.uk/pride/data/archive/2018/11/PXD010580/2_col_c2.raw |
| 2 / 4 | 17 | ftp://ftp.pride.ebi.ac.uk/pride/data/archive/2019/04/PXD010730/GZ01-Col-rep1.raw |

## **Supplementary Note 4: Preparation of proto-coccoliths**

Our approach to isolating coccolith vesicles targeted vesicles that contained a proto-coccolith, as the coccolith calcite is an easily visible marker for these organelles. Proto-coccoliths were prepared from cells grown in low calcium medium and in which the formation of coccoliths had been induced by adding CaCl_2_ solution to the culture at a concentration of 10 mM for 2 h. At harvesting, EDTA (10 mM final concentration) was added to dissolve already secreted coccoliths. The pelleted cells were washed with 100 mM Tris-HCl pH 8.5, 300 mM sorbitol, 200 mM NaCl, 30mM KCl, 10 mM MgCl2 and eventually resuspended in ice-cold lysis buffer (100 mM Tris-HCl pH 8.5 saturated with Na_2_CO_3_, 300 mM sorbitol, 200 mM NaCl, 30 mM KCl, 10 mM MgCl_2_, 10 mM dithiothreitol (DTT), 200 U DNAse I, 50 µM oryzalin, and cOmplete EDTA-Free Protease Inhibitor Mixture (Roche)). Cells were disrupted in 3 cycles at 2,000 psi and 4 °C using a French Press. Unbroken cells and aggregated material were removed by centrifugation at 200 g for 10 min. The supernatant was loaded on top of a sucrose two-step gradient (0.4 mL 70% and 1.6 mL 40% (w/v) sucrose solution). To generate the sucrose solutions, sucrose was dissolved in 100 mM Tris-HCl pH 8.5 saturated with Na_2_CO_3_. Gradients were centrifuged at 5,000 g for 1 h in a swing-out rotor. The material above the sucrose was discarded. The sucrose fraction was resuspended in 2 ml lysis buffer, loaded on top of a second sucrose step gradient (0.3 mL 70%, 0.3 mL 60%, and 0.3 mL 50% (w/v) sucrose solution), and centrifuged again. The entire gradient was fractioned from the top to the bottom using an Auto Densi-Flow fractionator (Labconco) and calcium was quantified in each fraction by ICP-OES. The two bottom fractions, showing the highest calcium content, were combined and analysed further. For electron microscopy analysis, all particulate material was pelleted and resuspended in 50 mM NH_4_HCO_3_. For determining whether or not the isolated proto-coccoliths are enveloped by a membrane, the material was (i) stained with the lipophilic dyes FM1-43 and NBD-ceramide, and (ii) analysed for protecting the calcite from dissolution by EDTA. Staining did not provide a clear answer, as control samples boiled in 2 % SDS solution to solubilize membranes still stained positive. For assaying dissolution protection, proto-coccoliths and isolated extracellular coccoliths were resuspended in 2 ml of lysis buffer. Proto-coccoliths in control samples were resuspended in lysis buffer supplemented with 2% (w/v) SDS, and heated to 99 °C for 1 h to solubilized the membranes. EDTA solution was added to all samples at room temperature to a final concentration of 10 mM. After 3 h incubation at room temperature, the samples were centrifuged at 10,000 g for 15 min, the pellet and supernatant were separated, and calcium was quantified in all fractions.

## **Supplementary Note 5: Proteomics sample preparation**

**Total protein for quantitative experiments:** Cultures of *E. huxleyi* were grown to mid-log phase (ca. 4 x 10^6^ cells / ml), and the volume of culture required for 2 x 10^8^ cells was harvested by centrifugation for 10 min at 2000g. The cell pellet was resuspended in 400 µl of extraction buffer (2% SDS, 100 mM HEPES pH 7.5, 2 mM DTT, 75 mM NaCl, 0.5% (w/v) PVPP, 1x Sigma cOmplete protease inhibitor cocktail. Samples were sonicated on ice four times at 30% duty with a pause of 30 s between each sonication, followed by centrifugation at 20,000 g and 4˚C for 30 min. Sample clean-up and digests were with a FASP/tryptic digest procedure, as described below.

**Coccosphere proteins:** Cultures of *E. huxleyi* grown in standard (10 mM) or low (0.1 mM) calcium containing media were harvested in early – mid log phase (2 x 10^6^ cells / ml), and transferred to fresh media by gentle centrifugation (1000g for 15 min) and resuspension (gentle pipetting with wide diameter tip) in 5 ml of media. EDTA was added to 10 mM, and the cultures incubated for 20 min. The dissolution of the coccosphere confirmed by microscopy in the case of calcifying cells. Cells were again gently pelleted, and the supernatant transferred to new tubes. After vortexing at maximum speed for 2 min, the supernatant was centrifuged at 20,000 g for 30 mins to removed insoluble material and cell debris. The samples were then loaded onto 10 kDa MWCO spin filters and the FASP/tryptic digest procedure followed as described below.

**Coccolith vesicle protein extracts:** Suspensions of proto-coccoliths were mixed with SDS an EDTA to the final concentrations of 2% (w/v) and 10 mM pH 8.0, respectively, incubated at 99 °C for 10 min before loading onto 30-kDa filter units (Microcon YM-10, Millipore). The extraction of peptides was performed according to a filter-aided sample preparation (FASP) protocol (Wisniewski et al., 2009). Proteins were digested with trypsin (Promega) and peptides desalted (Finisterre C18 SPE columns (WICOM International AG)), lyophilized and resuspended in 30 µl of 5% (v/v) acetonitrile and 2% (v/v) trifluoroacetic acid.

**Extraction of COPROs:** Freeze dried ESOM was resuspended in solutions, the composition of which depended on the enzyme later used for digestion: Trypsin, 6 M urea/2 M thiourea pH 8; GluC, 0.8M urea; LysC, 25 mM Tris-HCl, 1 mM EDTA, pH 8.5; AspN, 3.5 M urea; Trypsin + GluC, 0.8 M urea; Trypsin + LysC, 25 mM Tris-HCl, 1 mM EDTA, pH 8.5. To each sample, DTT was added to the final concentration of 5 mM followed by a 20 min incubation at 56°C. After cooling, iodoacetamide was added to a final concentration of 15 mM and samples were incubated in darkness for 15 min. Next samples were diluted, if necessary, with 10 mM Tris pH 8.0 such that the urea concentration was <1M, and 0.2 µg of the specific endoproteinase was added to each sample. Samples were incubated in 37°C overnight. In double digested samples the second enzyme was added after 5 h of incubation at 37°C. After incubation samples were acidified to pH 3 with 2% TFA and peptides were further desalted using ZipTips C18 columns (Merck Millipore). For in-gel digestion, each polyacrylamide gel slice was placed in a Protein LoBind tube (Eppendorf) and incubated three times for 20 min with a fresh solution of 200 µl of 50% acetonitrile in 50 mM NH_4_HCO_3_ followed by a 5 min incubation in 100% acetonitrile to remove the dye. Next, gel pieces were incubated in 100 µl 10 mM DTT, 50 mM NH_4_HCO_3_ for 30 min at 56°C, washed with 200 µl 100% acetonitrile for 5 min and incubated in 100 µl 55 mM iodoacetamide, 50 mM NH_4_HCO_3_ for 30 min in the dark. After removing the liquid, samples were washed with 200 µl 50% acetonitrile, 50 mM NH_4_HCO_3_ for 5 min and twice with 200 µl 100% acetonitrile for 5 min. Dry gel pieces were covered with precooled (4°C) trypsin solution (15 ng/µl in 50 mM NH_4_HCO_3_) and kept on ice for 30 min. Then the remaining liquid was removed and 50 µl of 50 mM NH_4_HCO_3_ was added to each sample before incubating overnight at 37°C. After digestion, the supernatant was collected into fresh tubes and gel pieces were incubated with 50 µl 30 % acetonitrile, 1 % TFA for 10 min followed by with 50 µl of 100% acetonitrile for 5 min to extract further peptides from the gel. All the extracts were pooled together, dried using a vacuum centrifuge and desalted using ZipTips C18 columns (Merck Millipore).

**FASP/tryptic digest procedure:** Samples were incubated for 40 min at room temperature in the presence of 100 mM. The sample was then loaded onto a 10 kDa MWCO spin filter (Microcon YM-10, Millipore) and centrifuged at 16,000 g for 15 min. The filtrate was discarded and the filter washed three times with 8 M urea, 0.1 M Tris pH 8.0 before addition of 30 mM idoacetamide in 200 µl of the same buffer and incubation for 30 min at room temperature in the dark. After centrifugation, the filter was further washed with 200 µl of 20 mM cysteine in 20 mM Tris pH 8.0 and then washed twice more with 100 µl of 20 mM Tris pH 8.0. The filter was incubated with 4 µg of trypsin (sequencing grade, Promega) in 80 µl of 10 mM Tris pH 8.0, 10 mM CaCl_2_ at 37 °C overnight. The peptides were eluted with three washes with 40 µl of 50 mM NH_4_HCO_3_ pH8.0 before being desalted on Finisterre C18 SPE columns (WICOM International AG) according to the manufacturer’s instructions. Dried peptides were stored at -80˚C.

**TMT-labelling:** Each TMT 10plex labelling reagent was resuspended in 40 µl of anhydrous ACN. 6 µl of label was added to 0.15 µg of purified peptides, resuspended in 100 mM triethylammonium bicarbonate and water was added to a final volume of 26 µl. The labelling reaction was allowed to proceed for 1 h at room temperature and then 2.5 µl of 5% (w/v) hydroxylamine was added and the reaction incubated for a further 15 min. The labelled peptides were then dried in a vacuum centrifuge and stored at -80˚C.

## **Supplementary Note 6: Other methodological details**

**SDS-PAGE.** For analysis of EDTA-soluble organic material by “Stains-All” staining ^18^, the samples were separated on 6% - 15 % gradient Schägger ^19^ gels. For analysis of EDTA-soluble organic material by silver staining, the samples were separated on 10 % or 12 % acrylamide Schägger gels. Equal amounts of sugar or protein were loaded in each lane. Protein concentrations were measured using a BCA protein assay method and sugar concentrations were measured using the phenol-sulfuric acid assay ^20^.

For preparation of whole cell protein extracts (TP), cells were decalcified by adding EDTA to the culture and pelleted by centrifugation. The cell pellet was resuspended in ice-cold 200 mM HEPES pH 7.5 and 200 mM NaCl buffer supplemented with cOmplete EDTA-Free Protease Inhibitor Mixture (Roche), and cells were disrupted on ice using sonication until the suspension appeared homogenous. To prepare membrane-bound protein extract (MP) and soluble protein extract (SP), the whole cell protein extract was ultracentrifugated at 100,000 g, 4 °C for 2 h and pellet (MP extract) and supernatant (SP extract) were separated. The pellet (MP extract) was washed with the initial buffer to remove residual soluble protein.

**Calcium quantification.** Calcium was quantified by inductively coupled plasma optical emission spectrometry (ICP-OES) on an Optima 8000 ICP-OES spectrometer (Perkin Elmer). Samples were dissolved in aqua regia at 50 °C for 20 min and calcium concentrations were calculated from measurements at 315 nm and 317 nm.

**Electron microscopy.** Scanning electron microscopy was performed on a Jeol JSM7500F FEG-SEM equipped with a Gatan ALTO 2500 cryo-preparation system (Gatan Inc., UK). For non-cryo imaging, samples were washed twice with 50 mM NH_4_HCO_3_ and air-dried on the sample holders. For cryoSEM imaging, samples were plunge-frozen in liquid nitrogen, transferred under vacuum into the preparation chamber (-130°C) and cryo-fractured. Subsequently samples were sputter coated with palladium under cryo-conditions. Samples were imaged at -130°C and an acceleration voltage of 1 – 3 kV.

## **Supplementary Note 7: Details of regulation of ion channels in the proteomics datasets, compared a previous study of gene expression using q-RT PCR** ^21^**.**

We used in silico searches with the primer sequences detailed by MacKinder *et al*. 2011 to see if we predict amplification of the same genes from the Emihu2 gene models as were targeted by MacKinder *et al*.. This analysis makes it clear which qPCR results from the MacKinder et al work can be compared with our data, and which cannot (Supplementary Table 11-14).

**Supplementary Table 11: Calcium transporter genes from the Emihu2 genome which were identified in the present proteomics studies or that we predict would be amplified by primers detailed in Table 1 of MacKinder *et al*. (2011).** If the gene is predicted to be amplified by the MacKinder primers, then the name of the primer is given, otherwise a cross indicates no amplification is predicted. In the former case it can be concluded that the q-RT PCR data from MacKinder *et al*. (2011) may be reporting on the expression of the Emihu2 gene in question. If the protein product was identified in one of the four proteomics datasets a tick is present in the appropriate column. The absence of the protein in the proteome data set is indicated by “-”. “Down in day” means down regulated in the light in the Light vs. Dark dataset. “Up in C-cells” means upregulated in C-cells in the C-cell vs. N-cell dataset. “Down in C-cells” means downregulated in that dataset.

| Emihu2 transcript or protein | Transporter type | MacKinder 2011 | Coccosphere | Down in light | Up in C-cells | Down in C-cells |
| --- | --- | --- | --- | --- | --- | --- |
| EhG30522.1 | CAX | CAX4 primer pair | - | - | - | - |
| EhG30522.2 | CAX | CAX4 primer pair | - | - | - | - |
| EhG18640.1 | CAX | CAX4 primer pair | - | - | - | - |
| EhG40126.1 (.15) | Na+/Ca2+K+ exchanger | x | 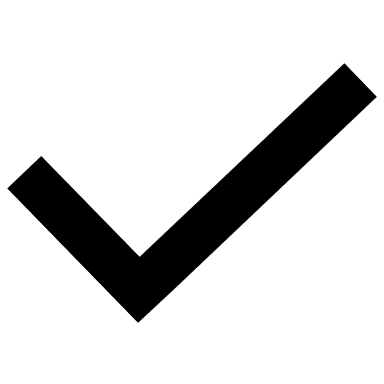 | 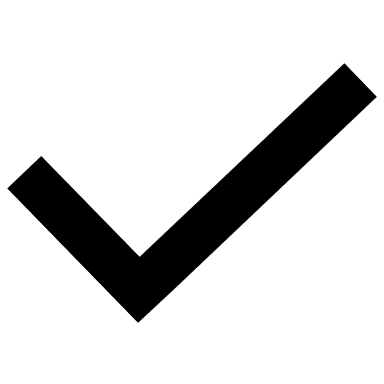 | - | 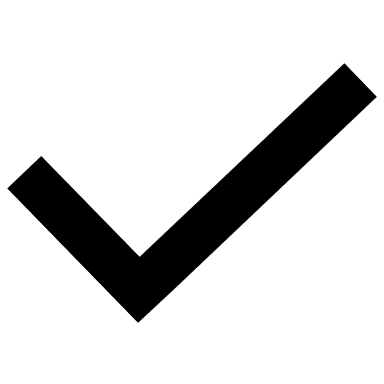 |
| EhG2170.2 | CAX | x | - | - | 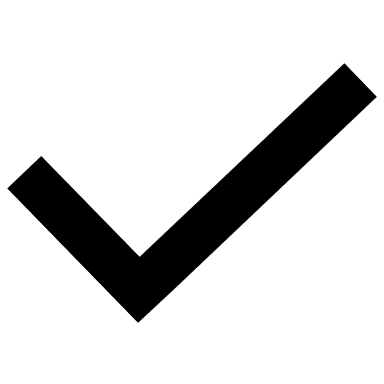 | - |
| EhG13953.3 | Na+/Ca2+K+ exchanger | x | - | - | - | 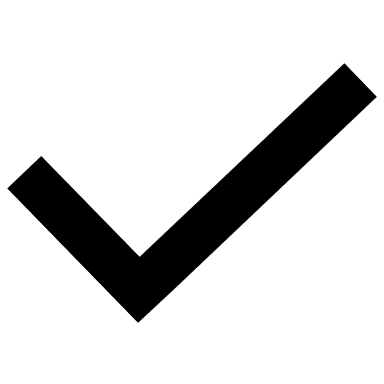 |
| EhG33932 (iso 1 - 3) | ECA2 | (ECA2 primer pair) | - | - | - | - |
| EhG853 (iso 1 – 4) | ECA2 | (ECA2 primer pair) | - | - | - | - |
| EhG20651.3 | ECA2 | x | - | - | - | 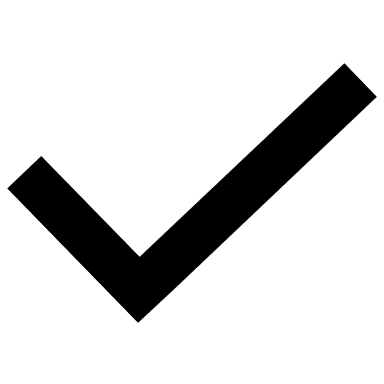 |

**Supplementary Table 12: AEL1 genes that would be amplified by the MacKinder *et al*. (2011) “AEL1” primers from Table 1, and those found in the present proteomics study.** If the gene is predicted to be amplified by the MacKinder primers, a tick is present in the Mackinder column and further details of the MacKinder results are provided. If the protein product was identified in one of the four proteomics datasets, a tick is present in the appropriate column. The absence of the protein in the proteome data set is indicated by “-”. “Down in day” means down regulated in the light in the Light vs. Dark dataset. “Up in C-cells” means upregulated in C-cells in the C-cell vs. N-cell dataset. “Down in C-cells” means downregulated in that dataset.

|  | MacKinder 2011 | Coccosphere | Down in day | Up in C-cells | Down in C-cells |
| --- | --- | --- | --- | --- | --- |
| EhG4408  (EhG4408.2 identified in proteomics) | 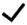  All isoforms 1-7 would be amplified.  2N-specific; up in C-cells vs N-cells | - | 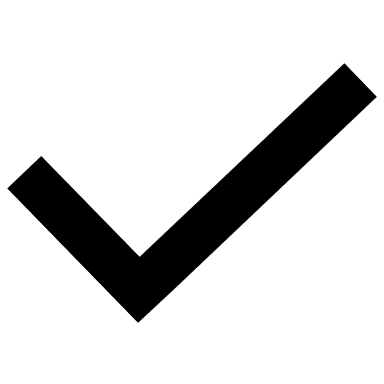 | - | 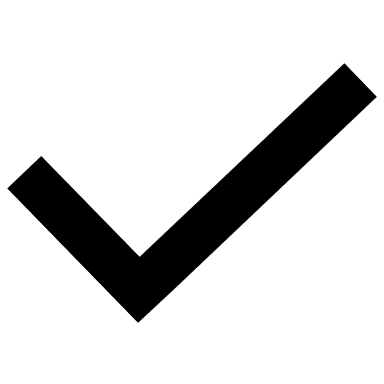 |
| EhG4885 (iso 1 – 3) | x | - | - | - | - |
| EhG31923.6 | x | - | - | - | 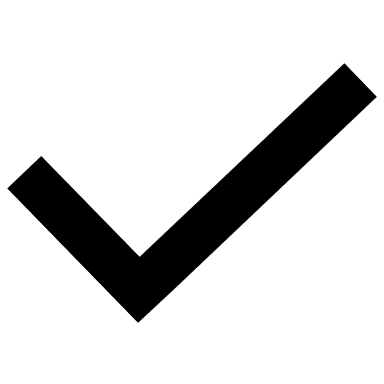 |
| EhG909.1 | x | - | - | - | 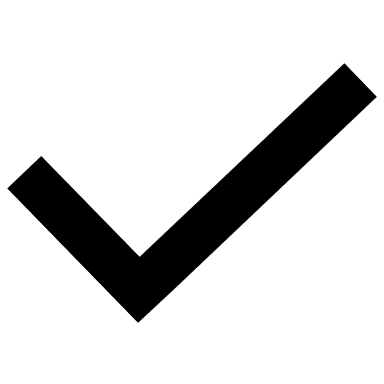 |
| EhG5929.10 | x | 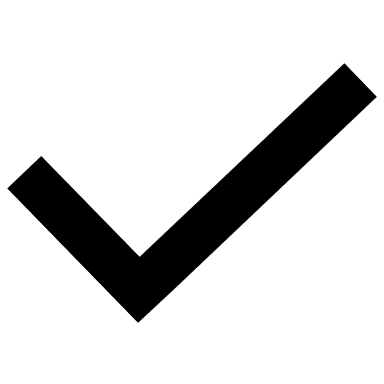 | 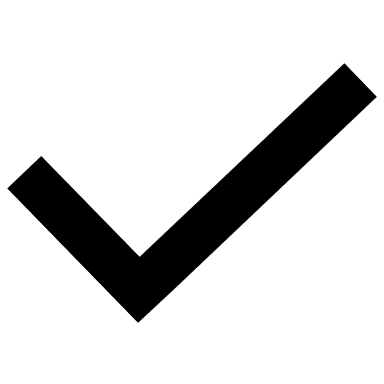 | - | 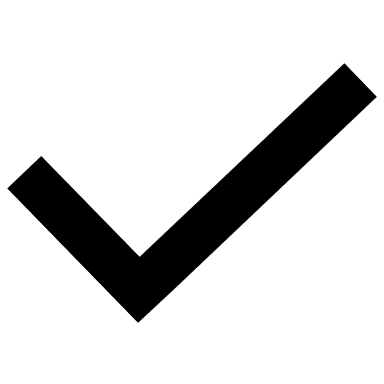 |

**Supplementary Table 13: V-type ATPase genes found in the Emihu2 genome and either identified in the present proteomics study or predicted to be amplified by the MacKinder *et al*. (2011) primers.** If they are predicted to be amplified there is a tick in the MacKinder 2011 column. If they were identified in a proteomics data set there is a tick in the relevant column. The absence of the protein in the proteome data set is indicated by "-".

|  | Subunit | MacKinder 2011 | CV | Down C-cells  Up N-cells |
| --- | --- | --- | --- | --- |
| EhG28591 | C | 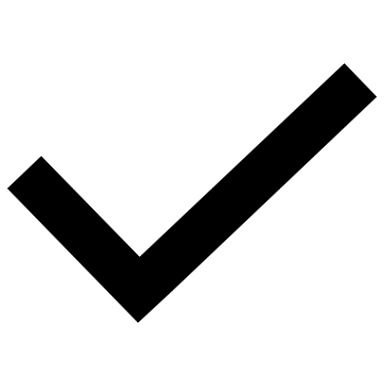 | - | - |
| EhG7733 | C | 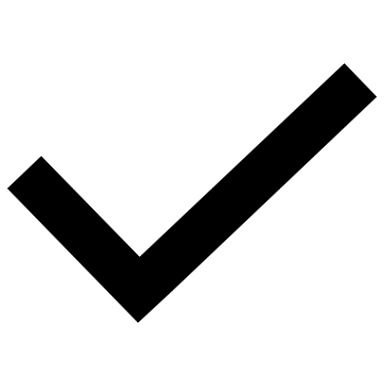 | - | - |
| EhG3936.1 | a/b | x | 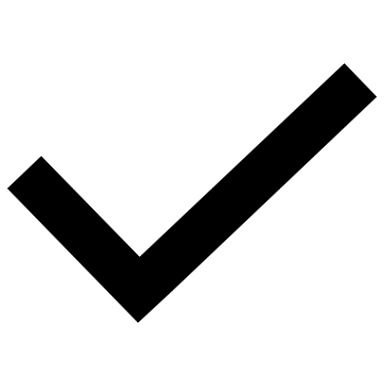 | 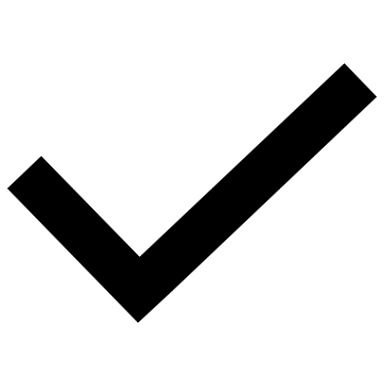 |
| EhG15825.1 | a/b | x | 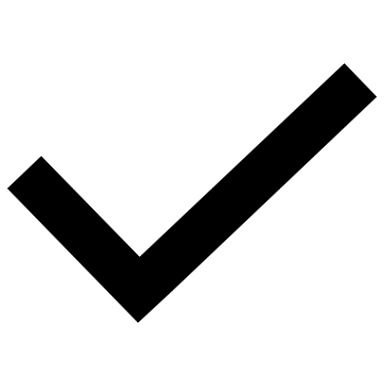 | - |
| EhG24295.1 | H | x | - | 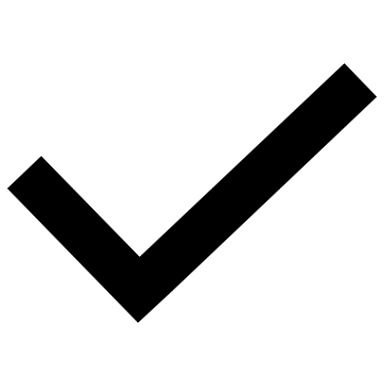 |
| EhG39357.3 | a/b | x | - | 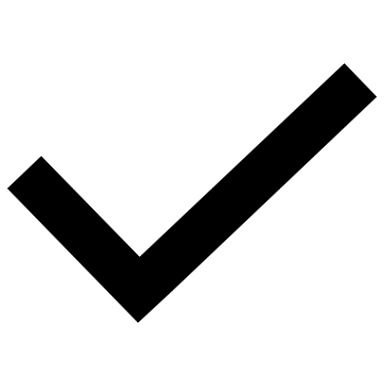 |
| EhG12509.8 | C | x | - | 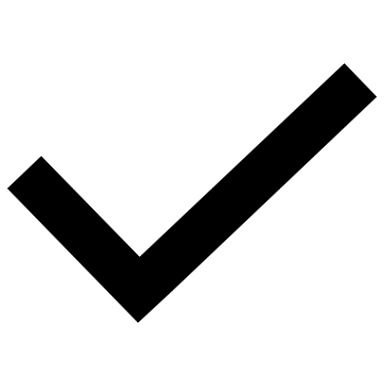 |
| EhG2867.8 | D | x | - | 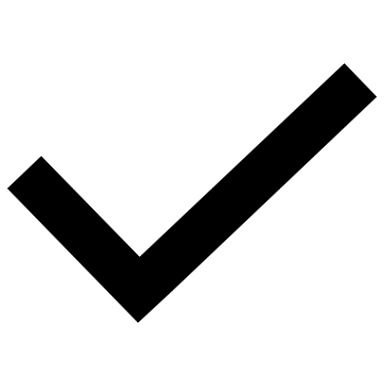 |
| EhG40709.2 | D | x | - | 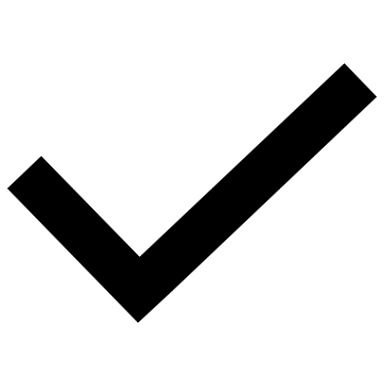 |
| EhG1300.1 | F | x | - | 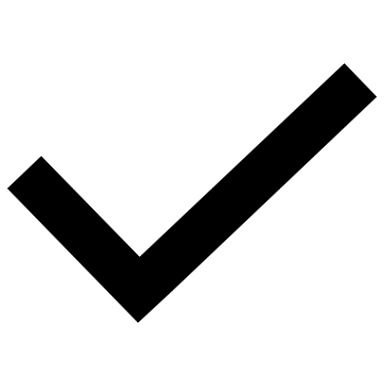 |
| EhG569.4 | C | x | - | 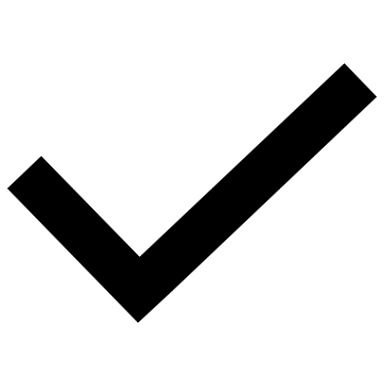 |
| EhG43802.2 | C/AC39 | x | - | 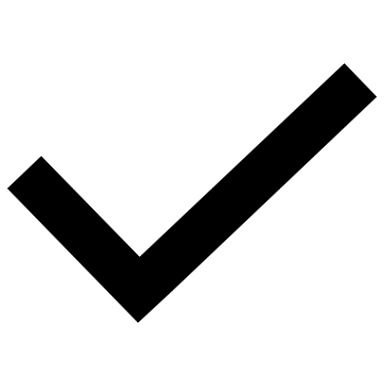 |
| EhG38205.7 | E | x | - | 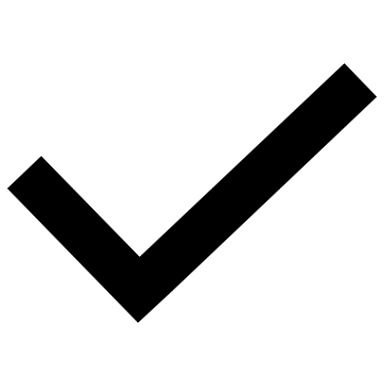 |
| EhG36849.1 | I | x | - | 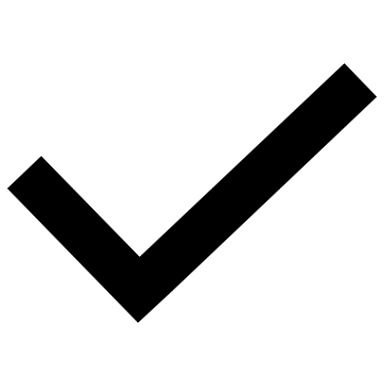 |
| EhG30295.1 | C | x | - | 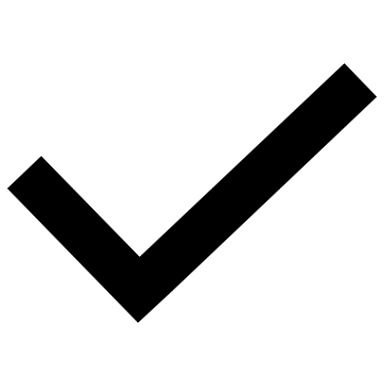 |
| EhG11411.7 | C | x | - | 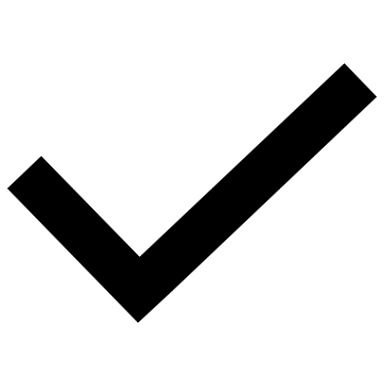 |
| EhG37694.1 | G | x | - | 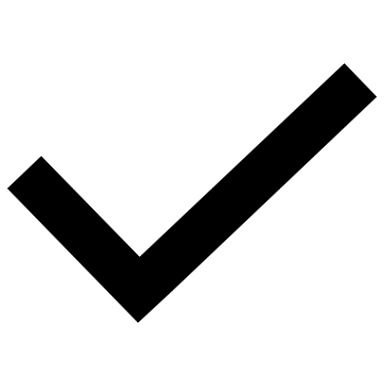 |

**Supplementary Table 14: CAX genes annotated in the Emihu2 genome.** Annotated as having “Calcium:proton antiporter activity” in EggNog annotation and high blast similarity to Emihu1 annotated CAX genes.

| **Locus** | **Possible allelic locus** |
| --- | --- |
| EhG2170 | EhG13465 |
| EhG18640 | EhG30522 |
| EhG2314 | EhG13328 |
| EhG22831 |  |
| EhG21902 | EhG19759 |
| Br7225 |  |
| Br7697 |  |
| Br8513 |  |
| Br27521 |  |
| Br33329 |  |
| Br33794 |  |

**Supplementary Note 8: Summary of filters and parameters used to define protein lists discussed in main text, figures in main text and when considering overlap between data sets.**

| **Dataset** | **Main text filters**  **(and supplementary figs)** | **Main Figure filers** | **Overlap filter** |
| --- | --- | --- | --- |
| Coccolith Vesicle | Min peptide probability = 0.95  Min protein probability = 0.99  Min unique peptides =2  Min replicates = 1 (out of 3)  Remove clear contaminants  Remove if only in N-cell data  Remove if distribution is the same across N-cell and C-cell fractions  [16 proteins] | As for main text | As for main text, but:  Min unique peptides = 1  [26 proteins] |
| COPROs | Min peptide probability = 0.95  Min protein probability = 0.99  Min unique peptides =2  Min replicates = 2 (out of 3)  Remove clear contaminants  [68 proteins] | As for main text except:  Min replicates = 3  No proteins older than Haptophyte age | As for main text |
| Coccosphere | Min peptide probability = 0.95  Min protein probability = 0.99  Min unique peptides =2  Min replicates = 3 (out of 4)  Remove clear intracellular contaminants  Remove Low-Ca proteins  [78 proteins] | Selected proteins of interest | As for main text, except:  Min replicates = 2  [96 proteins] |
| Recalcification | Min peptide probability = 0.95  Min protein probability = 0.99  Min unique peptides = 2  Min replicates = 2 (out of 3)  LIMMA adjusted p-value < 0.05 |  | Not included |
| Day vs Night | Min peptide probability = 0.95  Min protein probability = 0.99  Min unique peptides = 2  Min replicates = 2 (out of 3)  LIMMA adjusted p-value < 0.05  [DE = 142  Up in Day = 66  Down in Day = 76] | - | As for main text |
| C vs N-cells | Min peptide probability = 0.95  Min protein probability = 0.99  Min unique peptides = 2  Min replicates = 2 (out of 3)  LIMMA adjusted p-value < 0.05  [DE = 1291  Up in C-cells: 606  Down in C-Cells: 685] |  | As for main text |

# **References**

1. Burki, F., Roger, A. J., Brown, M. W. & Simpson, A. G. B. The new tree of Eukaryotes. *Trends Ecol. Evol.* **35**, 43-55 (2020).

2. Liu, H. *et al.* Extreme diversity in noncalcifying haptophytes explains a major pigment paradox in open oceans. *Proc. Natl. Acad. Sci.* **106**, 12803-12808 (2009).

3. Lin, Y., Chung, C., Gong, G. & Chiang, K. Diversity and abundance of haptophytes in the East China Sea. *Aquat. Microb. Ecol.* **72**, 227–240 (2014).

4. Gutowska, M. A. *et al.* Globally Important haptophyte algae use exogenous pyrimidine compounds more efficiently than thiamin. *MBio* **8**, e01459-17 (2017).

5. Skeffington, A. W. & Donath, A. ProminTools: Shedding light on proteins of unknown function in biomineralization with user friendly tools illustrated using mollusc shell matrix protein sequences. *PeerJ* **8**, e9852 (2020).

6. Johnson, L. S., Eddy, S. R. & Portugaly, E. Hidden Markov model speed heuristic and iterative HMM search procedure. *BMC Bioinform.* **11**, 431 (2010).

7. Sebé-Pedrós, A., Grau-Bové, X., Richards, T. A. & Ruiz-Trillo, I. Evolution and classification of myosins, a paneukaryotic whole-genome approach. *Genome Biol. Evol.* **6**, 290–305 (2014).

8. von Dassow, P. *et al.* Life-cycle modification in open oceans accounts for genome variability in a cosmopolitan phytoplankton. *ISME J.* **9**, 1365–1377 (2015).

9. Von Dassow, P. *et al.* Transcriptome analysis of functional differentiation between haploid and diploid cells of *Emiliania huxleyi*, a globally significant photosynthetic calcifying cell. *Genome Biol.* **10**, R114 (2009).

10. Almagro Armenteros, J. J. *et al.* SignalP 5.0 improves signal peptide predictions using deep neural networks. *Nat. Biotechnol.* **37**, 420–423 (2019).

11. Chin, C.-S. *et al.* Phased diploid genome assembly with single-molecule real-time sequencing. *Nat. Methods* **13**, 1050–1054 (2016).

12. Roach, M. J., Schmidt, S. A. & Borneman, A. R. Purge Haplotigs: Allelic contig reassignment for third-gen diploid genome assemblies. *BMC Bioinform.* **19**, 460 (2018).

13. Flynn, J. M. *et al.* RepeatModeler2 for automated genomic discovery of transposable element families. *Proc. Natl. Acad. Sci.* **117**, 9451–9457 (2020).

14. Bao, W., Kojima, K. K. & Kohany, O. Repbase update, a database of repetitive elements in eukaryotic genomes. *Mob. DNA* **6**, 11 (2015).

15. Huang, Y., Niu, B., Gao, Y., Fu, L. & Li, W. CD-HIT suite: a web server for clustering and comparing biological sequences. *Bioinformatics* **26**, 680–682 (2010).

16. Fürtauer, L., Pschenitschnigg, A., Scharkosi, H., Weckwerth, W. & Nägele, T. Combined multivariate analysis and machine learning reveals a predictive module of metabolic stress response in *Arabidopsis thaliana*. *Mol. Omi.* **14**, (2018).

17. Wu, G.-Z. *et al.* Control of retrograde signalling by protein import and cytosolic folding stress. *Nat. Plants* **5**, 525–538 (2019).

18. Campbell, K. P., MacLennan, D. H. & Jorgensen, A. O. Staining of the Ca^2+^-binding proteins, calsequestrin, calmodulin, troponin C, and S-100, with the cationic carbocyanine dye ‘Stains-all’. *J. Biol. Chem.* **258**, 11267–73 (1983).

19. Schägger, H. & von Jagow, G. Tricine-sodium dodecyl sulfate-polyacrylamide gel electrophoresis for the separation of proteins in the range from 1 to 100 kDa. *Anal. Biochem.* **166**, 368–79 (1987).

20. Fournier, E. Colorimetric quantification of carbohydrates. *Curr. Protoc. Food Anal. Chem.* **00**, E111-E118 (2001).

21. MacKinder, L. *et al.* Expression of biomineralization-related ion transport genes in *Emiliania huxleyi*. *Environ. Microbiol.* **13**, 3250–3265 (2011).
