# Supplementary material for: A joint proteomic and genomic investigation provides insights into the mechanism of calcification in coccolithophores
Source: Nat Commun. 2023 Jun 23;14:3749. doi: 10.1038/s41467-023-39336-1 (PMC10290126; doi:10.1038/s41467-023-39336-1)
Supplement: Supplementary file 3 — Description of Additional Supplementary Files [file 41467_2023_39336_MOESM3_ESM.pdf]

Description of Additional Supplementary Files

Supplementary Data 1
Description: Proteins identified by whole-cell proteomics whose abundance changes over a 6-hour time course of recalcification in noncalcifying and recalcifying cells.

Supplementary Data 2
Description: Candidate COccolith PROteins (COPROs)

Supplementary Data 3
Description: Candidate coccolith vesicle proteins identified by proteomic analysis of proto-coccolith samples.

Supplementary Data 4
Description: Candidate coccosphere proteins.

Supplementary Data 5 Description: Proteins identified by whole-cell proteomics that vary in abundance between C-cells and non-calcifying N-cells (CvsN dataset) and between day and night phases in C cells (Light vs. Dark dataset).

Supplementary Data 6
Description: Pfam domains that were over-represented in each orthogroups-age class compared to the entirety of the E. huxleyi proteome.
