# Supplementary material for: A joint proteomic and genomic investigation provides insights into the mechanism of calcification in coccolithophores
Source: Nat Commun. 2023 Jun 23;14:3749. doi: 10.1038/s41467-023-39336-1 (PMC10290126; doi:10.1038/s41467-023-39336-1)
Supplement: Supplementary file 10 — Reporting Summary [file 41467_2023_39336_MOESM10_ESM.pdf]

nature portfolio

Corresponding author(s): André Scheffel
Last updated by author(s): Apr 26, 2023

Reporting Summary

Nature Portfolio wishes to improve the reproducibility of the work that we publish. This form provides structure for consistency and transparency in reporting. For further information on Nature Portfolio policies, see our Editorial Policies and the Editorial Policy Checklist.

Statistics

For all statistical analyses, confirm that the following items are present in the figure legend, table legend, main text, or Methods section.

n/a	Confirmed	
☐	☒	The exact sample size (*n*) for each experimental group/condition, given as a discrete number and unit of measurement
☐	☒	A statement on whether measurements were taken from distinct samples or whether the same sample was measured repeatedly
☐	☒	The statistical test(s) used AND whether they are one- or two-sided<br>*Only common tests should be described solely by name; describe more complex techniques in the Methods section.*
☐	☒	A description of all covariates tested
☐	☒	A description of any assumptions or corrections, such as tests of normality and adjustment for multiple comparisons
☐	☒	A full description of the statistical parameters including central tendency (e.g. means) or other basic estimates (e.g. regression coefficient) AND variation (e.g. standard deviation) or associated estimates of uncertainty (e.g. confidence intervals)
☐	☒	For null hypothesis testing, the test statistic (e.g. *F*, *t*, *r*) with confidence intervals, effect sizes, degrees of freedom and *P* value noted<br>*Give P values as exact values whenever suitable.*
☒	☐	For Bayesian analysis, information on the choice of priors and Markov chain Monte Carlo settings
☒	☐	For hierarchical and complex designs, identification of the appropriate level for tests and full reporting of outcomes
☒	☐	Estimates of effect sizes (e.g. Cohen's *d*, Pearson's *r*), indicating how they were calculated

Our web collection on statistics for biologists contains articles on many of the points above.

Software and code

Policy information about availability of computer code

Data collection	MS acquisition: Xcalibur 2.1 (Thermo Scientific)
Data analysis	Tools used in this article (further details about command lines are found in Methods and/or Supplementary Information):<br>Genomic data: SMRTLink v7.0.1 (https://www.pacb.com)<br>Transcriptomic data: SMRTLink v6.0.0.47841 (https://www.pacb.com)<br>Falcon unzip v1.3.5 (Chin et al., 2016)<br>Purge Haplotigs v1.1.0 (Roach et al., 2018)<br>minimap2 v2.17-r943-dirty (Li et al 2018)<br>TAMA Collapse https://github.com/GenomeRIK/tama (Kuo et al., 2020)<br>getorf, EMBOSS (v6.6.0.0) https://www.bioinformatics.nl/cgi-bin/emboss/ (Rice et al., 2000)<br>gmst v1 http://topaz.gatech.edu/GeneMark/license_download.cgi (Tang, 2015)<br>TransDecoder v5.2 (Haas et al., 2013)<br>Hmmscan from HMMER3 3.2 (Eddy, 2009) with Pfam-A.hmm database (v32.0)<br>RepeatModeler v1.0.11 (https://www.repeatmasker.org/)<br>RepeatMasker v4.0.9 (https://www.repeatmasker.org/)<br>BRAKER v2.1.0 (Hoff et al., 2019)<br>gffcompare v0.10.6 (Pertea and Pertea, 2020)<br>PepNovo+ v 3.1 (Frank and Pevzner, 2005; Frank, 2009)<br>TPP version-5.2.0.1 http://tools.proteomecenter.org/ (Deutsch 2010)

CD-HIT v4.7 (https://github.com/weizhongli/cdhit) (Fu 2012)
BLAST+ v2.13 (ftp://ftp.ncbi.nlm.nih.gov/blast/executables/blast+)
limma v3.42.2 10.18129/B9.bioc.limma (Richie 2015)
qvalue v2.15.0 (https://github.com/StoreyLab/qvalue)
ggplot2 v3.3.2 (https://ggplot2.tidyverse.org/)
PRIDE Cluster algorithm: spectra-cluster-cli-1.1.2.jar https://github.com/spectra-cluster/spectra-cluster-cli (Griss et al., 2016)
InterProScan: InterPro 82.0 (Jones et al., 2014)
EggNog-Mapper v5.0 (Huerta-Cepas et al., 2019)
FinalORFs.R, this work, figshare (10.6084/m9.figshare.20464293.v1)
HECTAR v1.3 webtools.sb-roscoff.fr (Gschloessl et al., 2008)
pureseqTM v1.0 (Qing et al., 2019)
SignalP v 5.0, v6.0 https://services.healthtech.dtu.dk/service.php?SignalP (Teufel 2022)
Orthofinder v2.3.8 (Emms and Kelly, 2019)
qvalue_trunc.R (https://rdrr.io/github/StoreyLab/qvalue/src/R/qvalue_trunc.R).
ProminTools suit v03 https://hub.docker.com/r/biologistatsea/seprolyzer (Skeffington and Donath, 2020).
VLS2 (no version numbering exists) (Peng et al., 2006)
SEG (no version numbering exists) http://www.dbbm.fiocruz.br/cgc/seg.html (Wootton and Federhen, 1993)
fLPS (no version numbering exists) https://github.com/pmharrison/flps2 (Harrison, 2017)
Mascot 2.6.00 (Perkins et al., 1999)
Comet release 2018.01 rev. 4 (Eng et al., 2013)
Philosopher v4.0.0 / TMT-integrator framework with fragpipe (da Veiga Leprevost et al., 2020)
MS-fragger v3.1.1 (Kong et al., 2017)
BUSCO analysis v4.0.5, based on the eukaryote_odb10 orthogroup set

For manuscripts utilizing custom algorithms or software that are central to the research but not yet described in published literature, software must be made available to editors and reviewers. We strongly encourage code deposition in a community repository (e.g. GitHub). See the Nature Portfolio guidelines for submitting code & software for further information.

Data

Policy information about availability of data

All manuscripts must include a data availability statement. This statement should provide the following information, where applicable:

- Accession codes, unique identifiers, or web links for publicly available datasets
- A description of any restrictions on data availability
- For clinical datasets or third party data, please ensure that the statement adheres to our policy

Raw proteomics data were deposited with the ProteomeXchange Consortium via the PRIDE (Deutsch et al., 2020) partner repository under accessions PXD027059, PXD027440, PXD027481, PXD027501, PXD027515 and PXD027567. See Supplementary Table 7 for details. Raw PacBio sequencing data are available at the NCBI repository under BioProject accession number PRJNA789191. Annotated genome, transcriptome and proteome level data files are available at figshare (10.6084/m9.figshare.20464254). The JGI Emihu1 best proteins database is available at https://phycocosm.jgi.doe.gov/Emihu1. Arabidopsis data in supplementary figure 4 were downloaded from ftp://ftp.pride.ebi.ac.uk/pride/data/archive/2018/11/PXD010580/2_col_c2.raw and from ftp://ftp.pride.ebi.ac.uk/pride/data/archive/2019/04/PXD010730/GZ01-Col-rep1.raw. The Arabidopsis proteome data base used was Araport11 (https://www.arabidopsis.org/download_files/Proteins/Araport11_protein_lists/Araport11_pep_20220914.gz). The pfam A database v32 is available at http://ftp.ebi.ac.uk/pub/databases/Pfam/releases/Pfam32.0/Pfam-A.hmm.gz. The EukProt protein set used as a basis for phylostratigraphic reconstruction is available at figshare: https://doi.org/10.6084/m9.figshare.12417881.v3.

Human research participants

Policy information about studies involving human research participants and Sex and Gender in Research.

Reporting on sex and gender	not applicable
Population characteristics	not applicable
Recruitment	not applicable
Ethics oversight	not applicable

Note that full information on the approval of the study protocol must also be provided in the manuscript.

Field-specific reporting

Please select the one below that is the best fit for your research. If you are not sure, read the appropriate sections before making your selection.

[x] Life sciences [ ] Behavioural & social sciences [ ] Ecological, evolutionary & environmental sciences

For a reference copy of the document with all sections, see nature.com/documents/nr-reporting-summary-flat.pdf

Life sciences study design

All studies must disclose on these points even when the disclosure is negative.

Sample size	There were no sample size considerations in the sense often used in the biomedical literature (taking a representative sample of a large population). Samples were cultures, so measured variables are averages over millions of cells. There were samples size considerations regarding the number time points in a time-series. These were chosen to capture the dynamics of the physiological process under observation. There were also sample sizes considerations in terms of the number of replicates. These were as big as were practical given the work-flow constrains of the lab. No statistical methods were used to choose replicate number for two reasons. Firstly this was very novel research on a non-model organism where we had no expectations about the variance associated with experiments. Second, we only draw conclusions about proteins that may be involved in calcification. We accept that we will not have identified every proteins and do not draw conclusions from the absence of proteins so Type II error is of limited importance in this study. We consider it very unlikely that increasing the number of replicates would change our conclusions, although it might have revealed some more low abundance candidate proteins.
Data exclusions	When building the genome and transcriptome reads that map to the organella genomes of E. huxleyi were excluded because we wanted to study the nuclear encoded genes. Otherwise no data was excluded from the proteomics analyses until the final step of choosing lists of proteins of interest for each condition. Criteria such as identification in a minimum number of replicates, identification with a minimum number of peptides and presence of a signal peptide (depending on the dataset) were used to reduce false positive and derive lists of high confidence proteins. Details of these filters can be found in figure legends and Supplementary Note 8.
Replication	Data collection for the PacBio trasncriptome involved four biological replicates (independent cultures) per condition which were combined for sequencing since we aimed to develop a gene catalogue rather than compare conditions. All proteomic experiments involved replication, and identification in multiple replicates was used as a criterion for inclusion in final lists of proteins of interest. Exact numbers of replicates are stated in figure captions and methods. Use of number of replicates as a criteria is stated in figure captions and in Supplementary Note 8.
Randomization	Where cultures were assigned to experimental groups (e.g. standard vs. low calcium) this was done by random selection by the researcher. There were no visual distinctions between the cultures, so this is not a possible source of bias.
Blinding	No explicit blinding strategy was performed in our study. This is because the methods used for sample comparison (LC-MS/MS, SDS-PAGE, HPAEC-PAD) are not affected by user bias. The same settings were used for each sample, so no user bias is possible. The LC-MS/MS runs were de facto blinded since the operator was unaware of the meaning of the sample codes.

Reporting for specific materials, systems and methods

We require information from authors about some types of materials, experimental systems and methods used in many studies. Here, indicate whether each material, system or method listed is relevant to your study. If you are not sure if a list item applies to your research, read the appropriate section before selecting a response.

Materials & experimental systems

n/a	Involved in the study	
☒	☐	Antibodies
☒	☐	Eukaryotic cell lines
☒	☐	Palaeontology and archaeology
☐	☒	Animals and other organisms
☒	☐	Clinical data
☒	☐	Dual use research of concern

Methods

n/a	Involved in the study	
☒	☐	ChIP-seq
☒	☐	Flow cytometry
☒	☐	MRI-based neuroimaging

Animals and other research organisms

Policy information about studies involving animals; ARRIVE guidelines recommended for reporting animal research, and Sex and Gender in Research

Laboratory animals	Emiliania huxleyi strain AWI1516, derived from CCMP1516 and available from the Alfred Wegener Institute. CCMP2090 is available from NCMA at Bigelow Laboratory (https://ncma.bigelow.org).
Wild animals	This study did not involve wild animals.
Reporting on sex	Not appropriate to this sudy of algal biology.
Field-collected samples	Study did not involve samples collected from the field.
Ethics oversight	No ethical approval or guidance was required for work on algae.

Note that full information on the approval of the study protocol must also be provided in the manuscript.
